# Highly Efficient Asymmetric [3+2] Cycloaddition Promoted by Chiral Aziridine-Functionalized Organophosphorus Compounds

**DOI:** 10.3390/molecules29143283

**Published:** 2024-07-11

**Authors:** Julia Szymańska, Michał Rachwalski, Adam M. Pieczonka

**Affiliations:** 1 Department of Organic and Applied Chemistry, Faculty of Chemistry, University of Lodz, Tamka 12, PL-91-403 Lodz, Poland; julia.szymanska@edu.uni.lodz.pl (J.S.); adam.pieczonka@chemia.uni.lodz.pl (A.M.P.); 2 Doctoral School of Exact and Natural Sciences, University of Lodz, Matejki 21/23, PL-90-237 Lodz, Poland

**Keywords:** asymmetric cycloaddition, aziridines, chiral ligands/catalysts, enantioselective synthesis

## Abstract

The asymmetric [3+2] cycloaddition of azomethine ylides generated from the corresponding imino ester-to-*trans*-β-nitrostyrene catalysis by chiral aziridine-containing phosphines and phosphine oxides is described. Of the sixteen stereoisomers that could be formed as a result of the title reaction, three were formed, two of which were obtained in an enantiomerically enriched or pure form, and one in a racemic form. One of the products underwent epimerization under basic reaction conditions.

## 1. Introduction

**Figure 1:**
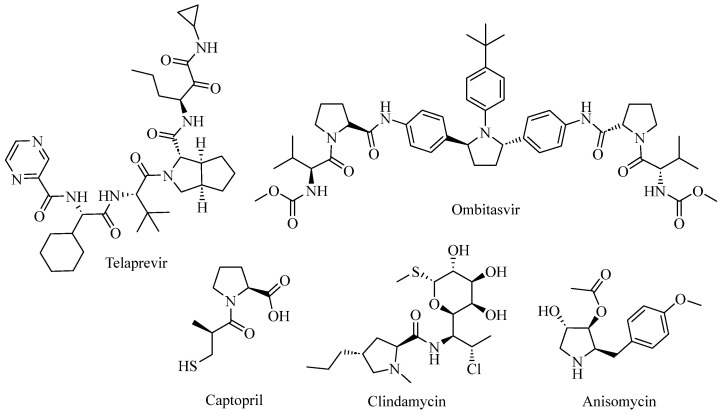
Examples of substances containing a pyrrolidine motif.

Asymmetric synthesis including organocatalysis is still one of the most important and intensively researched methodologies for creating new carbon–carbon bonds [1]. Within the above methodology, new trends are emerging, including, for example, photocatalysis [2] and asymmetric synthesis using free radicals [3]. The latter can be used in the synthesis of chiral amines and their derivatives [4], as well as in reactions of the enantioselective formation of carbon–carbon bonds, e.g., the alpha-oxyamination of aldehydes [5]. Among the very wide variety of asymmetric reactions, stereodifferentiating pericyclic reactions [6], including cycloadditions [7], deserve a special mention. Among the products of asymmetric cycloaddition reactions, chiral systems containing a pyrrolidine ring often play a key role in biological and pharmacological research [8]. They may have a wide spectrum of activities—antibacterial, cytotoxic, antifungal, etc. [9]. Some examples of substances containing a pyrrolidine motif available on the pharmaceutical market are Telaprevir, which is an antiviral agent used in the treatment of chronic hepatitis C virus infection [10]; Ombitasvir is also used as a strong inhibitor of SARS-CoV-2 [10]; Captopril is commonly used in the treatment of hypertension; and Clindamycin and Anisomycin have antibacterial properties [11] (Figure 1).

Based on our experience in the field of asymmetric synthesis using heterorganic ligands and organocatalysts [12], and taking into account the significant importance of chiral pyrrolidine systems in several areas of life and science [10,11], we decided to carry out the asymmetric [3+2] cycloaddition of azomethine ylides to nitrostyrene [8] using chiral, optically pure organophosphorus derivatives of aziridines as catalysts, namely phosphines [13], phosphine oxides [14], and aziridine-containing imines [15,16]. The purpose of this study was also to expand the scope of applicability of the chiral catalysts we had previously obtained. It should also be emphasized that the asymmetric [3+2] cycloaddition reaction catalyzed by copper (II) complexes is a chemical transformation that has been quite significantly exploited in the literature [17,18].

## 2. Results and Discussion

### 2.1. Synthesis of Chiral Catalysts and the Starting Materials

**Figure 2:**
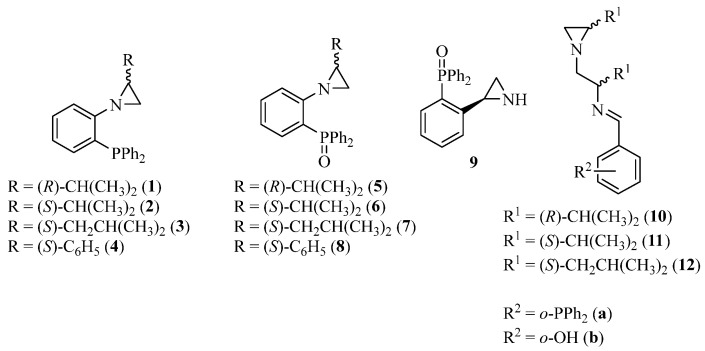
Chiral aziridine-containing catalysts **1**–**12**.

We started from the synthesis of the corresponding chiral catalysts **1**–**12** (Figure 2) (Table 1). Aziridine phosphines **1**–**4** were obtained from the corresponding phosphine oxides **5**–**8** using triethoxysilane and titanium (IV) isopropoxide [13]. Aziridine phosphine oxides **5**–**8** were synthesized starting with *o*-bromoanisole and diphenylphosphinic chloride as described previously [14]. In turn, imines **10**–**12** were obtained using the previously described protocols [15,16]. Finally, phosphine oxide-bearing NH-aziridine subunit **9** was prepared with (*S*)-2-phenylaziridine and diphenylphosphinic chloride in the presence of sec-BuLi [19].

**Figure 3:**
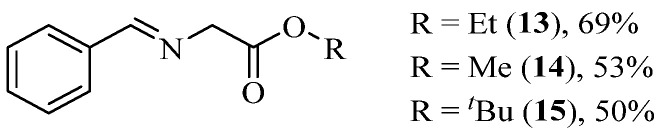
Structures of imino esters **13**–**15**.

Secondly, the corresponding imino esters **13**–**15** (Figure 3) being the substrates used for the in situ generation of azomethine ylides were prepared with the appropriate glycine esters and benzaldehyde in the presence of triethylamine according to the literature’s general protocol [20].

### 2.2. Asymmetric [3+2] Cycloaddition Reactions

**Scheme 1:**
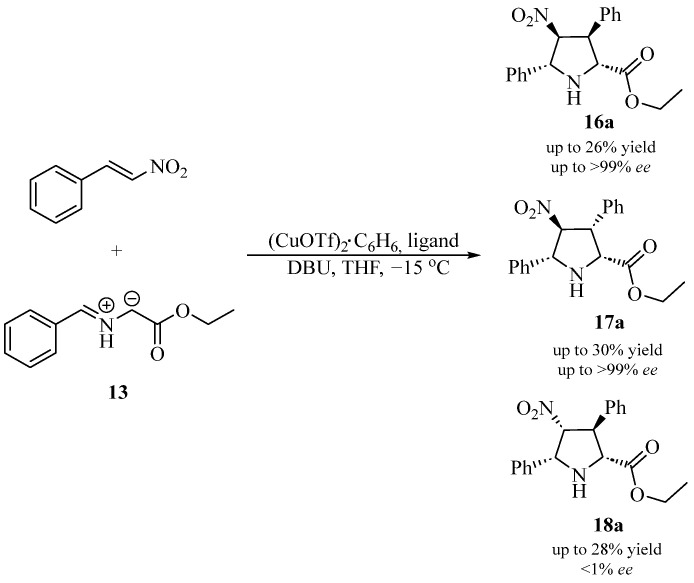
Asymmetric [3+2] cycloaddition in the presence of aziridine ligands **1**–**12**.

All the aziridine derivatives **1**–**12** were examined for catalytic activity in the asymmetric [3+2] cycloaddition reaction occurring between *trans*-*β*-nitrostyrene and ethyl imino ester **13** (Scheme 1). These reactions were catalyzed using an in situ generated catalytic system consisting of copper triflate, a chiral ligand, and DBU as a basic additive. After the appropriate purification of crude mixtures by column chromatography, three diastereomeric products **16**–**18** were obtained. Two of them were identified based on the literature data as products *exo* **17a** and *endo* **18a** (Scheme 1) [21,22]. However, compound **16a** has not been described in the literature. The effectiveness of the ligands was determined based on the analysis of the optical purity of the obtained products using the HPLC method using a column with chiral support. The results are summarized in Table 2.

The analysis of the results showed that the use of structurally similar aziridine ligands led to similar outcomes. The highest chemical yield of asymmetric [3+2] cycloaddition, up to 71%, was achieved using ligands with an imine group; however, the products were formed without significant diastereoselectivity. The aziridine phosphine ligands resulted in the formation of diastereomeric products in a similar ratio, with only aziridine phosphine ligand **2** shifting the equilibrium towards the formation of product *exo* **17a,** with additional excellent enantioselectivity (up to 98% *ee*). In all the cases, products **16a** and **17a** were formed in enantiomerically enriched forms, while compound **18a** always formed racemic mixtures. Unexpectedly, the use of ligand **9** containing an NH-aziridine group led to the formation of racemic product *endo* **18a** predominating over products **16a** and **17a**.

**Scheme 2:**
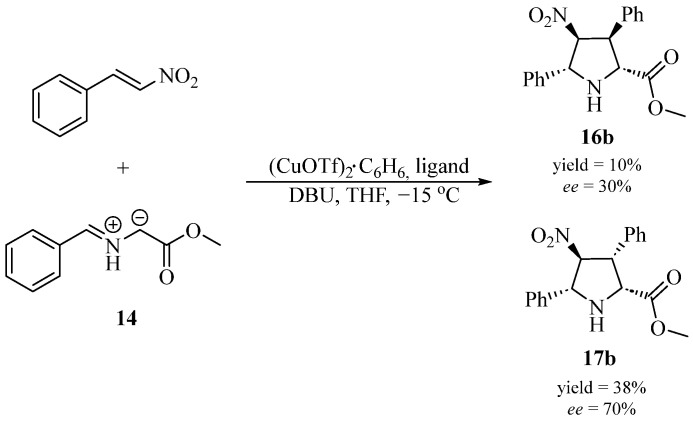
Asymmetric [3+2] cycloaddition of methyl imino ester **14**.

In the next stage, it was decided to conduct an asymmetric [3+2] cycloaddition reaction, but instead of ethyl imino ester **13**, methyl imino ester **14** was used. The second substrate and the other reaction conditions remained unchanged (Scheme 2) (Table 3). (*S*)-Isopropyl aziridine phosphine oxide **6** was used as the ligand. In this reaction, two diastereomeric products were acquired and also identified based on the literature-described ^1^H-NMR spectra of the *exo* **17b** product [21], which was formed with a 70% enantiomeric excess. Based on the ^1^H-NMR spectrum, the second of the formed diastereomers was also identified as the *4-epi-endo* **16b** product [8]. However, the third *endo* product formed in the previous reaction with ethyl imino ester **13** was not obtained this time.

**Scheme 3:**
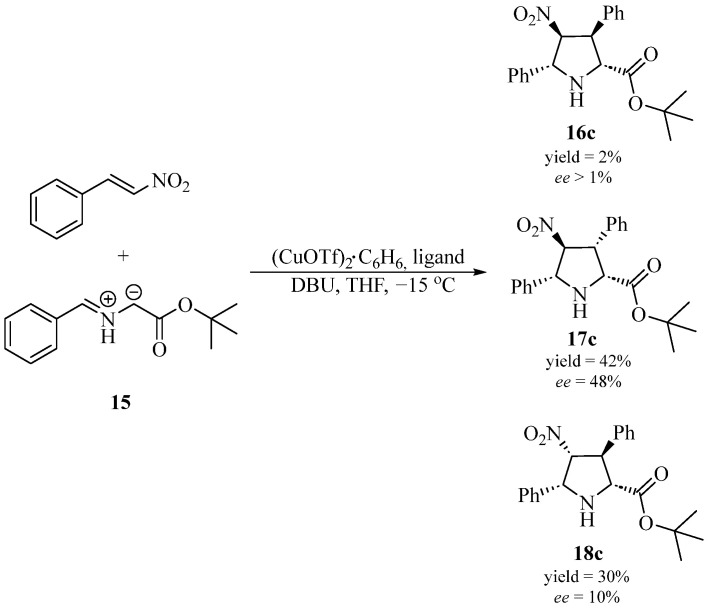
Asymmetric [3+2] cycloaddition of *tert*-butyl imino ester **15**.

In the next approach, the reaction was carried out using *tert*-butyl ester **15** as the substrate (Scheme 3). In this reaction, three diastereomeric products **16**–**18** were obtained again, and their configuration was determined based on the literature data as *4-epi-endo* **16c**, *exo* **17c**, and *endo* **18c** [8]. Interestingly, in this reaction, product **16c** was formed in a small racemic amount (Table 4).

In summary, after identifying all three products, it was concluded that the reaction proceeded according to a concerted mechanism, resulting initially in the formation of two products with *exo* **17** and *endo* **18** configurations. The formation of an additional diastereomeric product **16** should be impossible when *trans*-*β*-nitrostyrene is used as a substrate. Therefore, based on the literature reports, it is believed that product **18** undergoes epimerization under basic conditions (the aziridine ring exhibits a basic character). Epimerization involves a change in the configuration of a substituent at a single stereogenic center and is a process described in the literature [8] for both methyl and *tert*-butyl imino esters (e.g., involving triethylamine). It is assumed that the aziridine ligands act as chiral bases, causing the selective epimerization of product **18** to product **16**. Methyl derivatives are the most susceptible to this change; no *endo* product was observed because the entirety underwent epimerization. However, *tert*-butyl derivatives are the most resistant to epimerization; only trace amounts of product **16c** were observed. The differences in the quantity of product formed during the epimerization process may result from the steric hindrance present in the individual compounds. 

**Scheme 4:**
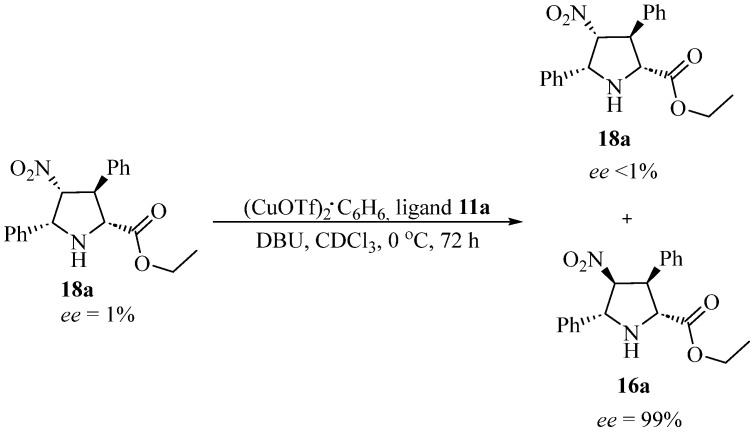
Base-promoted epimerization of the product **18a**.

To confirm the epimerization process, an additional experiment was conducted involving the reaction of pure product **18a** with the in situ generated catalytic system consisting of aziridine chiral ligand **11a**, a copper triflate, and DBU (Scheme 4). The reaction was conducted under analogous conditions to the cycloaddition reaction. This test confirmed that the mixture contained the *4-epi-endo* product **16a** along with the initial *endo* compound **18a** in a ratio of 0.6:1.0, demonstrating that the formation of product **16a** occurred under the influence of the utilized catalytic system and confirming the previously assumed theory of epimerization.

**Figure 4:**
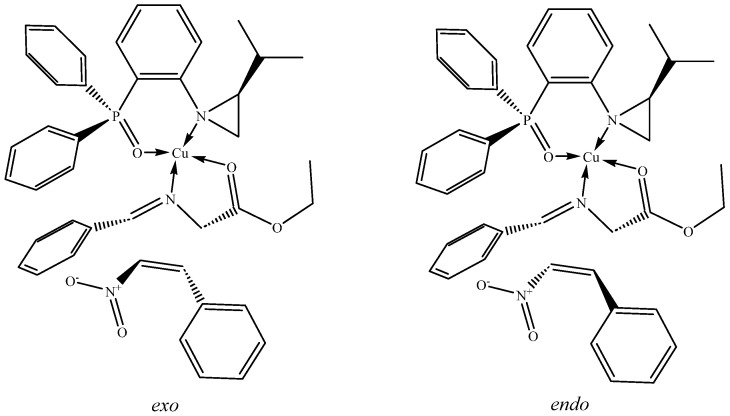
Tentative transition state model.

Finally, we attempted to explain the stereochemical course of the titular reaction by proposing a transition state model (Figure 4). It follows that coordination through the oxygen and nitrogen atoms of the imino ester and the phosphorus and nitrogen atoms of the chiral ligand makes β-nitrostyrene approachable from both the *endo* and *exo* sides. The (*S*)-isopropyl moiety on aziridine causes there to be less space around the Cu atom, which makes the system more strained, and thus causes higher enantioselectivity in comparison with that of the (*R*)-isopropyl substituent. Moreover, the absolute configuration of the resulting products does not change due to changes in the ligand configuration because the resulting intermediate complex is quite rigid and cannot change the configuration during the approach of nitrostyrene. During the formation of the intermediate complex, it is possible to arrange the substituents in such a way that the ethoxy group of the imino ester is very closely oriented to the isopropyl group, which may cause additional interactions in such an energetically favorable conformation, and therefore the enantioselectivity is higher for ethyl esters. Additionally, when it comes to the phenomenon of epimerization, in the space, the NO_2_ group is positioned further away from the ester group due to repulsive electrostatic interactions; therefore, the *exo* and *4*-*epi-endo* products are preferred.

## 3. Materials and Methods

### 3.1. General Information

All reagents were used as obtained from commercial suppliers unless otherwise noted. The corresponding chiral catalysts **1**–**12**, exactly, aziridine phosphines [13], aziridine phosphine oxides [14], phosphine oxide containing NH-aziridine subunit [19], and aziridine-containing imines [15,16], were prepared according to a literature report. Also, imino esters **13**–**15**, being substrates for in situ generation of azomethine ylides, were obtained using a general protocol [20]. NMR spectra for the solutions in deuterated chloroform (CDCl_3_) were recorded at 600 MHz (^1^H NMR) and 150 MHz (^13^C NMR) with a Bruker Avance III spectrometer (Bruker, Billerica, MA, USA) using the solvent as an internal standard. The following abbreviations were used to describe the NMR spectra: δ, chemical shift (ppm); *J,* coupling constants (Hz); s, singlet; br.s, broad singlet; d, doublet; dd, double-doublet; t, triplet; q, quartet; and m, multiplet. Column chromatography was performed with silica gel using a solvent mixture of hexane/ethyl acetate as eluents (9:1). The enantiomeric excess (*ee*) values were determined by high-performance liquid chromatography (HPLC) with a chiral packed column (Chiralcel OD-H) using hexane and isopropanol as the mobile phase. 

### 3.2. Asymmetric [3+2] Cycloaddition Reaction Catalyzed by Aziridine Derivatives ***1**–**12***—General Procedure

A copper triflate (CuOTf)_2_·C_6_H_6_ (0.1 mmol) and ligand (0.1 mmol) were placed in a flask, the whole mixture was cooled to 0 °C, and then DBU (12 µL) and anhydrous THF (4 mL) were added. The catalytic system was used for 4 h at 0 °C. The mixture was cooled to −15 °C, and imino ester (0.5 mmol) was added and stirred for 10 min, after which trans-β-nitrostyrene (0.5 mmol) was added. The resulting mixture was stirred for 48 h at a low temperature, and then the solvent was evaporated in vacuo. The crude products were separated via column chromatography with silica gel (hexane–ethyl acetate 9:1). All the aziridine derivatives **1**–**12** were examined for catalytic activity in the asymmetric [3+2] cycloaddition of ethyl imino ester **13**. In the asymmetric [3+2] cycloaddition of methyl imino ester **14** and *tert*-butyl imino ester **15,** only catalyst **6** was examined. Copies of all the NMR spectra are included in Supplementary Materials.

#### Characterization of Compounds **16a**–**c**, **17a**–**c**, and **18a**,**c**

(2*R*,3*S*,4*S*,5*R*)-Ethyl 4-Nitro-3,5-diphenylpyrrolidine-2-carboxylate (*4-epi-endo*) **16a**; yellow sticky oil, 45 mg, 26%. ^1^H NMR (600 MHz, CDCl_3_) δ: 7.58–7.56 (m, 2H), 7.45–7.43 (m, 2H), 7.39–7.33 (m, 4H), 7.29–7.28 (m, 2H), 5.16 (dd, *J* = 3.7 Hz, *J* = 7.6 Hz, 1H), 5.10 (d, *J* = 3.7 Hz, 1H), 4.70 (d, *J* = 3.7 Hz, 1H), 4.23 (q, *J* = 7.1 Hz, 2H), 4.07–4.05 (m, 1H), 2.93 (br.s, 1H), 1.24 (t, *J* = 7.1 Hz, 3H). ^13^C{^1^H} NMR (150 MHz, CDCl_3_) δ: 172.5, 140.2, 133.2, 129.0, 128.8, 128.4, 128.2, 126.8, 96.5, 65.8, 63.3, 61.6, 52.6, 14.0. Anal. Calcd. for C_19_H_20_N_2_O_4_: C, 67.05; H, 5.92; N, 8.23; O, 18.80; Found: C, 66.85; H, 5.75; N, 8.09; O, 19.31.

(2*R*,3*R*,4*S*,5*R*)-Ethyl 4-Nitro-3,5-diphenylpyrrolidine-2-carboxylate (*exo*) **17a** [21]; yellow sticky oil, 51 mg, 30%. ^1^H NMR (600 MHz, CDCl_3_) δ: 7.60–7.59 (m, 2H), 7.47–7.41 (m, 3H), 7.34–7.29 (m, 5H), 5.24 (t, *J* = 8.2 Hz, 1H), 4.79 (br.s, 1H), 4.51 (d, *J* = 9.0 Hz, 1H), 4.42 (t, *J* = 8.2 Hz, 1H), 3.89–3.83 (m, 1H), 3.76–3.70 (m, 1H), 2.77 (br.s, 1H), 0.85 (t, *J* = 7.1 Hz, 3H). ^13^C{^1^H} NMR (150 MHz, CDCl_3_) δ: 171.4, 137.7, 136.2, 129.1, 129.0, 128.8, 128.1, 128.0, 126.9, 95.3, 67.6, 64.2, 61.1, 53.8, 13.5. 

(2*R*,3*S*,4*R*,5*R*)-Ethyl 4-Nitro-3,5-diphenylpyrrolidine-2-carboxylate (*endo*) **18a** [22]; yellow sticky oil, 48 mg, 28%. ^1^H NMR (600 MHz, CDCl_3_) δ: 7.45–7.33 (m, 10H), 5.33 (dd, *J* = 3.8 Hz, *J* = 6.5 Hz, 1H), 4.96 (br.s, 1H), 4.37–4.25 (m, 2H), 4.24 (dd, *J* = 3.8 Hz, *J* = 7.5 Hz, 1H), 4.16–4.15 (m, 1H), 3.39 (br.s, 1H),1.29 (t, *J* = 7.5 Hz, 3H). ^13^C{^1^H} NMR (150 MHz, CDCl_3_) δ: 171.3, 138.7, 134.6, 129.3, 128.8, 128.1, 127.6, 126.5, 97.1, 67.8, 67.6, 61.7, 55.6, 14.1.

(2*R*,3*S*,4*S*,5*R*)-Methyl 4-Nitro-3,5-diphenylpyrrolidine-2-carboxylate (*4-epi-endo*) **16b** [8]; yellow sticky oil, 17mg, 10%. ^1^H NMR (600 MHz, CDCl_3_) δ: 7.56–7.55 (m, 2H), 7.45–7.31 (m, 6H), 7.28–7.27 (m, 2H), 5.15 (dd, *J* = 3.7 Hz, *J* = 7.6 Hz, 1H), 5.09 (d, *J* = 3.7 Hz, 1H), 4.72 (d, *J* = 9.1 Hz, 1H), 4.08 (d, *J* = 8.5 Hz, 1H), 3.78 (s, 3H), 2.92 (br.s, 1H). ^13^C{^1^H} NMR (150 MHz, CDCl_3_) δ: 172.9, 140.2, 133.1, 129.0, 128.9, 128.4, 128.1, 126.8, 96.6, 65.7, 63.1, 52.6, 52.4.

(2*R*,3*R*,4*S*,5*R*)-Methyl 4-Nitro-3,5-diphenylpyrrolidine-2-carboxylate (*exo*) **17b** [21]; yellow sticky oil, 65 mg, 38%. ^1^H NMR (600 MHz, CDCl_3_) δ: 7.60–7.58 (m, 2H), 7.46–7.39 (m, 3H), 7.35–7.26 (m, 5H), 5.24 (t, *J* = 8.3 Hz, 1H), 4.79 (d, *J* = 7.3 Hz, 1H), 4.53 (d, *J* = 8.5 Hz, 1H), 4.41 (t, *J* = 8.3 Hz, 1H), 3.32 (s, 3H), 2.78 (br.s, 1H). ^13^C{^1^H} NMR (150 MHz, CDCl_3_) δ: 171.9, 137.6, 135.9, 129.1, 129.0, 128.8, 128.2, 127.9, 126.9, 95.1, 67.6, 64.3, 53.8, 51.8.

(2*R*,3*S*,4*S*,5*R*)-*Tert*-butyl 4-Nitro-3,5-diphenylpyrrolidine-2-carboxylate (*4-epi-endo*) **16c** [8]; yellow sticky oil, 4 mg, 2%. ^1^H NMR (600 MHz, CDCl_3_) δ: 7.35–7.34 (m, 2H), 7.34–7.33 (m, 2H), 7.33–7.32 (m, 4H), 7.29–7.27 (m, 2H), 5.14 (dd, *J* = 3.7 Hz, *J* = 8.2 Hz, 1H), 5.07 (d, *J* = 3.7 Hz, 1H), 4.57 (d, *J* = 9.3 Hz, 1H), 3.91 (t, *J* = 8.2 Hz, 1H), 2.84 (br.s, 1H), 1.39 (s, 9H). ^13^C{^1^H} NMR (150 MHz, CDCl_3_) δ: 171.7, 140.2, 133.4, 129.0, 128.7, 128.4, 128.3, 126.8, 96.7, 82.2, 65.9, 64.1, 53.5, 27.9.

(2*R*,3*R*,4*S*,5*R*)-*Tert*-butyl 4-Nitro-3,5-diphenylpyrrolidine-2-carboxylate (*exo*) **17c** [8]; yellow sticky oil, 72 mg, 42%. ^1^H NMR (600 MHz, CDCl_3_) δ: 7.40–7.39 (m, 2H), 7.34–7.29 (m, 8H), 5.17 (t, *J* = 7.7 Hz, 1H), 4.75 (d, *J* = 6.7 Hz, 1H), 4.43 (d, *J* = 8.9 Hz, 1H), 4.35–4.32 (m, 1H), 2.74 (br.s, 1H), 1.08 (s, 9H). ^13^C{^1^H} NMR (150 MHz, CDCl_3_) δ: 170.0, 137.8, 137.2, 129.1, 128.9, 128.8, 128.4, 128.0, 126.9, 96.0, 81.9, 67.4, 64.6, 53.4, 27.4.

(2*R*,3*S*,4*R*,5*R*)-*Tert*-butyl 4-Nitro-3,5-diphenylpyrrolidine-2-carboxylate (*endo*) **18c** [8]; yellow sticky oil, 51 mg, 30%. ^1^H NMR (600 MHz, CDCl_3_) δ: 7.37–7.28 (m, 10H), 5.33–5.32 (m, 1H), 4.94 (br.s, 1H), 4.14–4.12 (m, 1H), 4.03 (br.s, 1H), 3.36 (br.s, 1H), 1.48 (s, 9H). ^13^C{^1^H} NMR (150 MHz, CDCl_3_) δ: 170.5, 138.8, 134.7, 129.2, 128.8, 128.7, 128.0, 127.6, 126.5, 97.1, 82.5, 68.2, 67.8, 56.1, 28.0.

## 4. Conclusions

In summary, the obtained ligands containing both arylphosphine or arylphosphinyl groups, an imine group, and an optically pure chiral aziridine ring that proved to be efficient catalysts for the asymmetric [3+2] cycloaddition reaction. The use of the aforementioned catalysts led to the formation of three products, *4-epi-endo* **16**, *exo* **17**, and *endo* **18**, of which products *4-epi-endo* **16** and *exo* **17** typically were formed in enantiomerically enriched forms. Meanwhile, the product *endo* **18** always formed as a racemic mixture. Compounds **17** and **18** were the products of a reaction occurring according to a concerted mechanism, whereas the formation of product **16** can be explained by the epimerization of product **18** under catalytic reaction conditions, which was confirmed by an independently conducted experiment. The differences in the quantity of the product formed during the epimerization process may result from the steric hindrance present in the different compounds. 

## Figures and Tables

**Table 1:** Catalysts **1**–**12**.

Ligand No.	Yield [%]	[α]_D_ [C 0.5] ^1^	*ee* [%] ^2^
**1**	55	−60.2	>99
**2**	60	+21.3	>99
**3**	53	+32.6	>99
**4**	52	+25.8	>99
**5**	67	−9.6	>99
**6**	56	+23.2	>99
**7**	54	+24.8	>99
**8**	62	+58.1	>99
**9**	65	+18.0	>99
**10a**	94	−4.8	>99
**10b**	87	−18.0	>99
**11a**	99	+4.8	>99
**12a**	97	+4.8	>99

^1^ In chloroform; ^2^ determined by chiral HPLC.

**Table 2:** Asymmetric [3+2] cycloaddition of ethyl imino ester **13**.

Ligand No.	Ligand	Yield [%]	Ratio 16a/17a/18a	*ee* [%] 16a	*ee* [%] 17a	*ee* [%] 18a
**(*R*)-1**	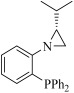	52	1.0/4.5/2.2	67	63	1
**(*S*)-2**	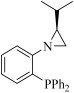	41	1.4/7.6/1.0	74	98	8
**(*S*)-3**	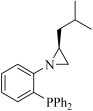	62	1.0/3.1/3.6	95	95	5
**(*S*)-4**	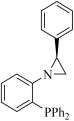	57	1.0/3.2/4.1	67	97	6
**(*R*)-5**	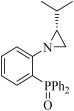	51	1.0/1.5/1.3	78	>99	2
**(*S*)-6**	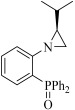	59	1.1/1.2/1.0	95	99	6
**(*S*)-7**	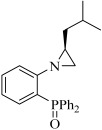	54	1.0/2.4/1.6	94	99	4
**(*S*)-8**	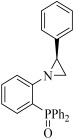	54	1.0/2.3/1.9	21	88	2
**(*S*)-9**	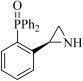	45	1.0/2.8/3.9	26	96	4
**(*R,R*)-10a**	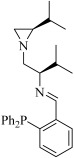	66	1.0/1.2/1.5	95	42	1
**(*R,R*)-10b**	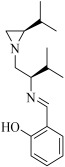	71	1.2/1.5/1.0	83	72	4
**(*S,S*)-11a**	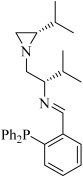	37	1.3/1.4/1.0	>99	94	1
**(*S,S*)-12a**	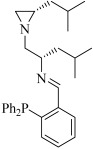	64	1.0/1.2/1.0	3	98	5

**Table 3:** Asymmetric [3+2] cycloaddition of methyl imino ester **14**.

Ligand No.	Ligand	Yield [%]	Ratio 16b/17b	*ee* [%] 16b	*ee* [%] 17b
**(*S*)-6**	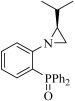	48	1.0/3.8	30	70

**Table 4:** Asymmetric [3+2] cycloaddition of *tert*-butyl imino ester **15**.

Ligand No.	Ligand	Yield [%]	Ratio 16c/17c/18c	*ee* [%] 16c	*ee* [%] 17c	*ee* [%] 18c
**(*S*)-6**	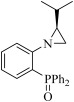	74	1.0/19.0/13.0	>1	48	10

## Data Availability

All the data are deposited on the hard-drive.

## Supplementary Materials

The following supporting information can be downloaded at: https://www.mdpi.com/article/10.3390/molecules29143283/s1, Section S1: Copies of NMR spectra, and Section S2: HPLC chromatograms.

## References

[1] Xiang S.-H., Tan B. (2020). Advances in organocatalysis over the last 10 years. Nat. Commun..

[2] Mancheño O.G., Waser M. (2023). Recent Developments and Trends in Asymmetric Organocatalysis. Eur. J. Org. Chem..

[3] Nagib D.A. (2022). Asymmetric Catalysis in Radical Chemistry. Chem. Rev..

[4] Friestad G.K. (2014). Control of Asymmetry in the Radical Addition Approach to Chiral Amine Synthesis. Top. Curr. Chem..

[5] Bertelsen S., Nielsen M., Jørgensen K.A. (2007). Radicals in Asymmetric Organocatalysis. Angew. Chem. Int. Ed..

[6] Das K.K., Kumar P., Ghorai D., Mondal B., Panda S. (2022). Organoboron Compounds Towards Asymmetric Pericyclic Reaction; Exploitation to Bioactive Molecule Synthesis. Asian J. Org. Chem..

[7] Held F.E., Tsogoeva S.B. (2016). Asymmetric cycloaddition reactions catalyzed by bifunctional thiourea and squaramide organocatalysts: Recent advances. Catal. Sci. Technol..

[8] Li J.-Y., Kim H.Y., Oh K. (2015). Brucine Diol-Catalyzed Asymmetric Synthesis of endo-Pyrrolidines: The Mechanistic Dichotomy of Imino Esters. Org. Lett..

[9] Adrio J., Carretero J.C. (2019). Stereochemical diversity in pyrrolidine synthesis by catalytic asymmetric 1,3-dipolar cycloaddition of azomethine ylides. Chem. Commun..

[10] Jeelan Basha N., Basavarajaiah S.M., Shyamsunder K. (2022). Therapeutic potential of pyrrole and pyrrolidine analogs: An update. Mol. Divers..

[11] Poyraz S., Döndaş H.A., Döndaş N.Y., Sansano J.M. (2023). Recent insights about pyrrolidine core skeletons in pharmacology. Front. Pharmacol..

[12] Leśniak S., Rachwalski M., Pieczonka A.M. (2014). Optically pure aziridinyl ligands as useful catalysts in the stereocontrolled synthesis. Curr. Org. Chem..

[13] Buchcic A., Zawisza A., Leśniak S., Rachwalski M. (2020). Asymmetric Friedel-Crafts alkylation of indoles catalyzed by chiral aziridine-phosphines. Catalysts.

[14] Wujkowska Z., Zawisza A., Leśniak S., Rachwalski M. (2019). Phosphinoyl-aziridines as a new class of chiral catalysts for enanti-oselective Michael addition. Tetrahedron.

[15] Buchcic-Szychowska A., Adamczyk J., Marciniak L., Pieczonka A.M., Zawisza A., Leśniak S., Rachwalski M. (2021). Efficient asymmetric Simmons-Smith cyclopropanation and diethylzinc addition to aldehydes promoted by enantiomeric aziridine-phosphines. Catalysts.

[16] Pieczonka A.M., Marciniak L., Rachwalski M., Leśniak S. (2020). Enantiodivergent aldol condensation in the presence of aziridine/acid/water systems. Symmetry.

[17] Rohilla S., Shah S., Singh V.K. (2023). Copper-Catalyzed Asymmetric Propargylic [3+2] Cycloaddition: Synthesis of Enantioenriched Dihydrofuro[3,2-c]coumarins and its Quinolinone and Thiocoumarin Analogues. Org. Lett..

[18] Chen Y., Zhao J.-Q., Zhang Y.-P., Zhou M.-Q., Zhang X.-M., Yuan W.-C. (2023). Copper-Catalyzed Asymmetric Dearomative [3+2] Cycloaddition of Nitroheteroarenes with Azomethines. Molecules.

[19] Buchcic A., Zawisza A., Leśniak S., Adamczyk J., Pieczonka A.M., Rachwalski M. (2019). Enantioselecive Mannich Reaction Promoted by Chiral Phosphinoyl-Aziridines. Catalysts.

[20] Zeng W., Chen G.-Y., Zhou Y.-G., Li Y.-X. (2007). Hydrogen Bond Directed Reversal of Enantioselectivity. J. Am. Chem. Soc..

[21] Bai X.-F., Song T., Xu Z., Xia C.-G., Huang W.-S., Xu L.-W. (2015). Aromatic Amide-Derived Non-Biaryl Atropisomers as Highly Efficient Ligands in Silver-Catalyzed Asymmetric Cycloaddition Reactions. Angew. Chem. Int. Ed..

[22] Kumar A., Gupta G., Srivastava S. (2010). Diversity oriented synthesis of pyrroli-dines via natural carbohydrate solid acid catalyst. J. Comb. Chem..

