# Supplementary material for: Highly Efficient Asymmetric [3+2] Cycloaddition Promoted by Chiral Aziridine-Functionalized Organophosphorus Compounds
Source: Molecules. 2024 Jul 11;29(14):3283. doi: 10.3390/molecules29143283 (PMC11279481; doi:10.3390/molecules29143283)
Supplement: Supplementary file 1 [file molecules-29-03283-s001.zip › molecules-3055527-supplementary.pdf]

SUPPORTING INFORMATION

Highly Efficient Asymmetric [3+2] Cycloaddition Promoted by Chiral Aziridine-Functionalized Organophosphorus Compounds

Julia Szymańska[1,2], Michał Rachwalski[1]*, Adam M. Pieczonka[1]

[1]University of Lodz, Faculty of Chemistry, Department of Organic and Applied Chemistry, Tamka 12, PL-91-403, Lodz, Poland

[2]University of Lodz, Doctoral School of Exact and Natural Sciences, Matejki 21/23, PL-90-237, Lodz, Poland

Table of contents

1. Copies of ^{1}H and ^{13}C NMR spectra **S2**

2. HPLC chromatograms **S10**

1. Copies of ^{1}H and ^{13}C NMR spectra

The ^{1}H and ^{13}C NMR of **16a**

^{1}H NMR (600 MHz, $CDCl_3$):

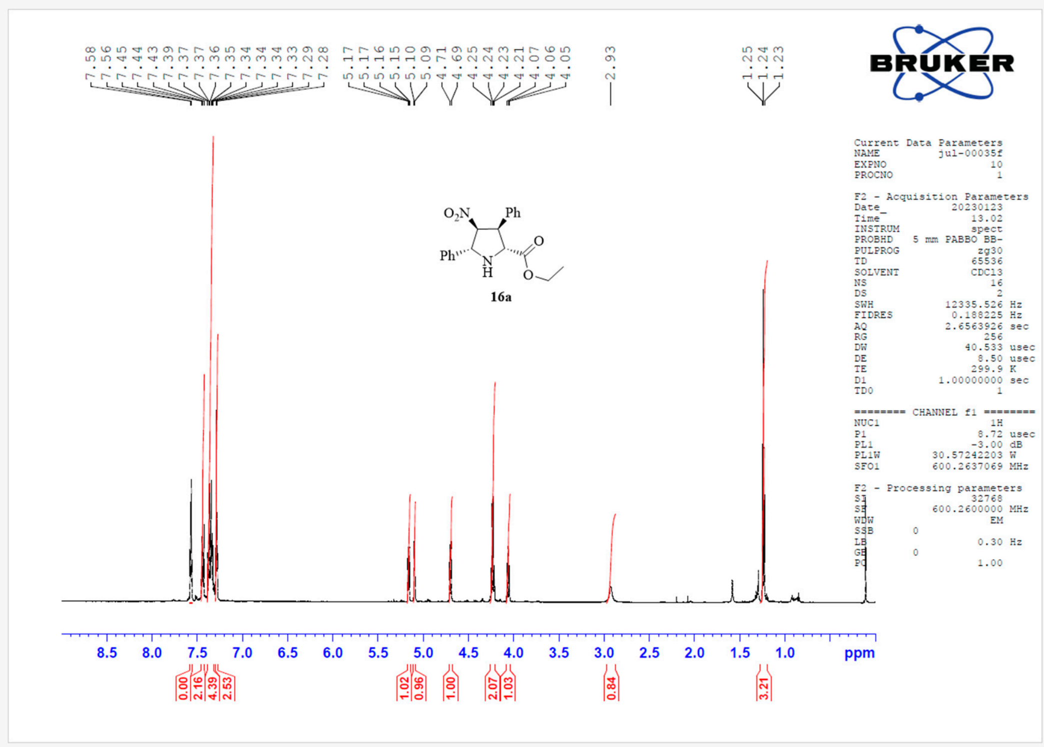

^{13}C NMR (150 MHz, $CDCl_3$):

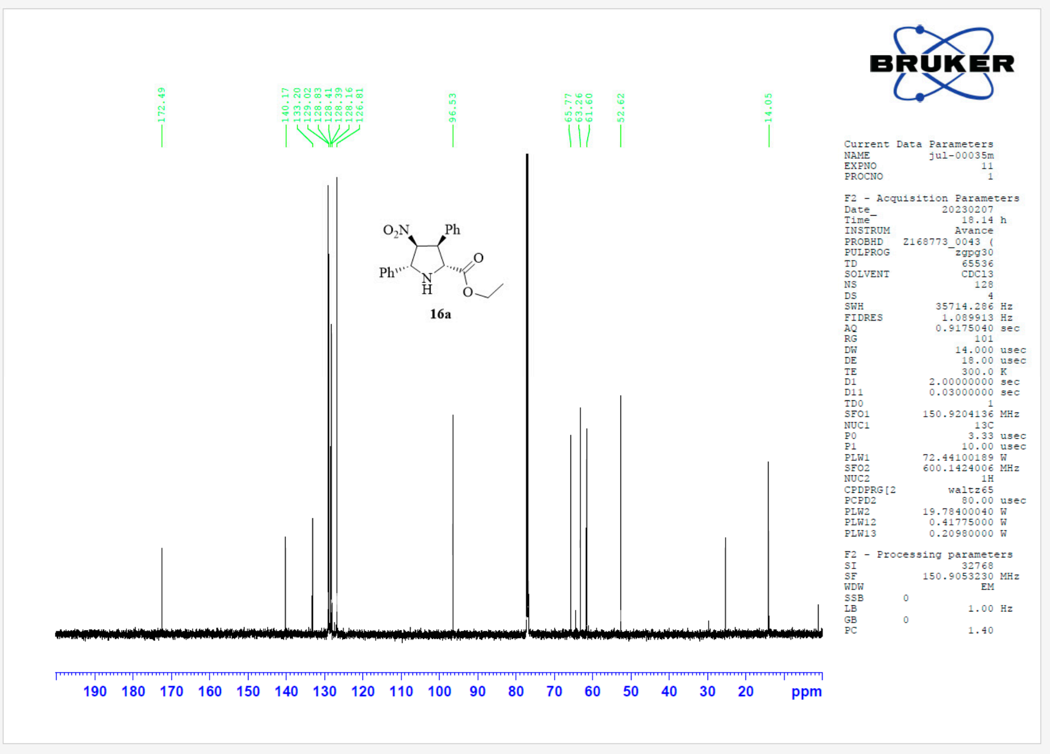

The ^{1}H and ^{13}C NMR of **17a**

^{1}H NMR (600 MHz, CDCl$_3$):

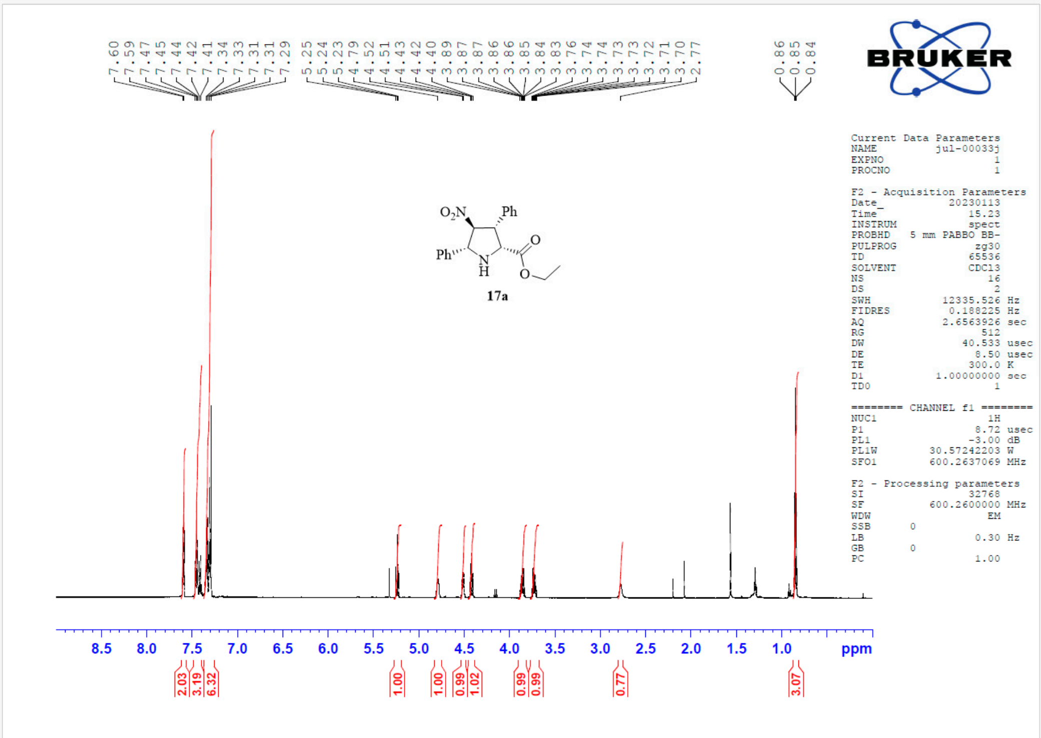

^{13}C NMR (150 MHz, CDCl$_3$):

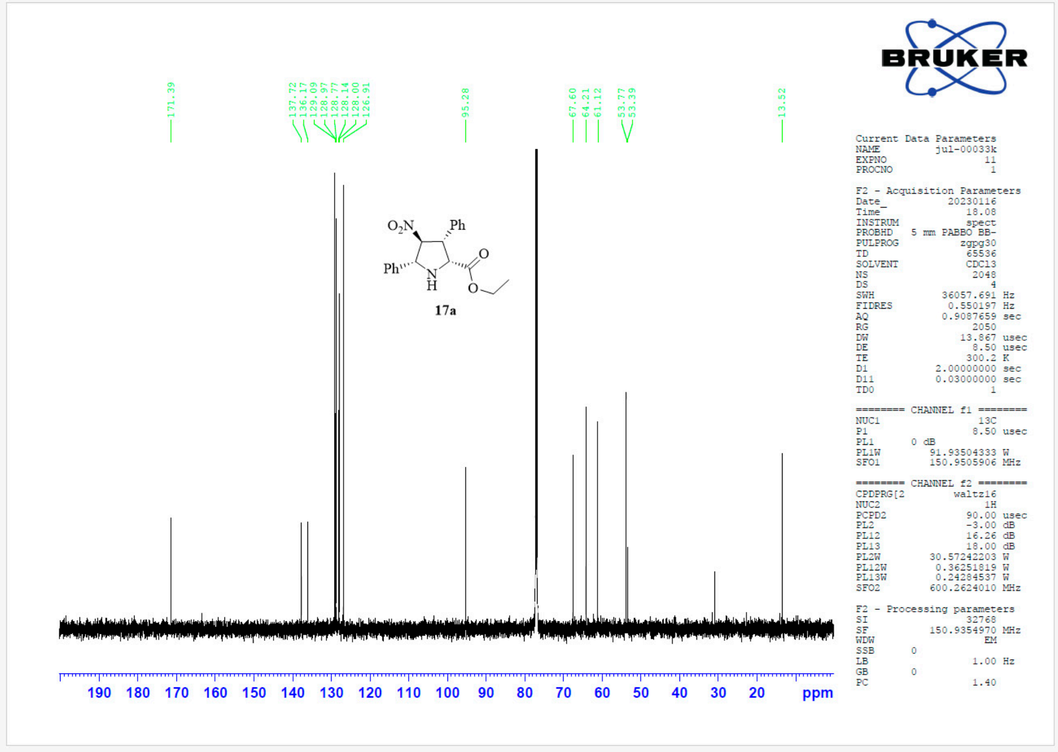

The ^{1}H and ^{13}C NMR of **18a**

^{1}H NMR (600 MHz, $CDCl_3$):

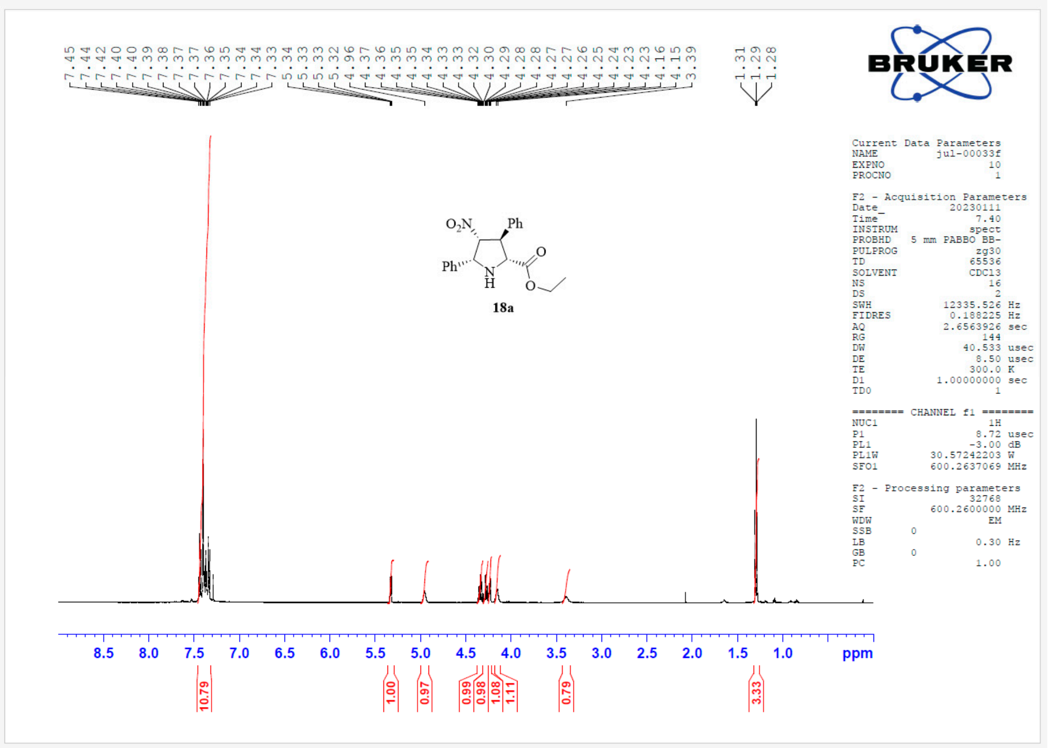

^{13}C NMR (150 MHz, $CDCl_3$):

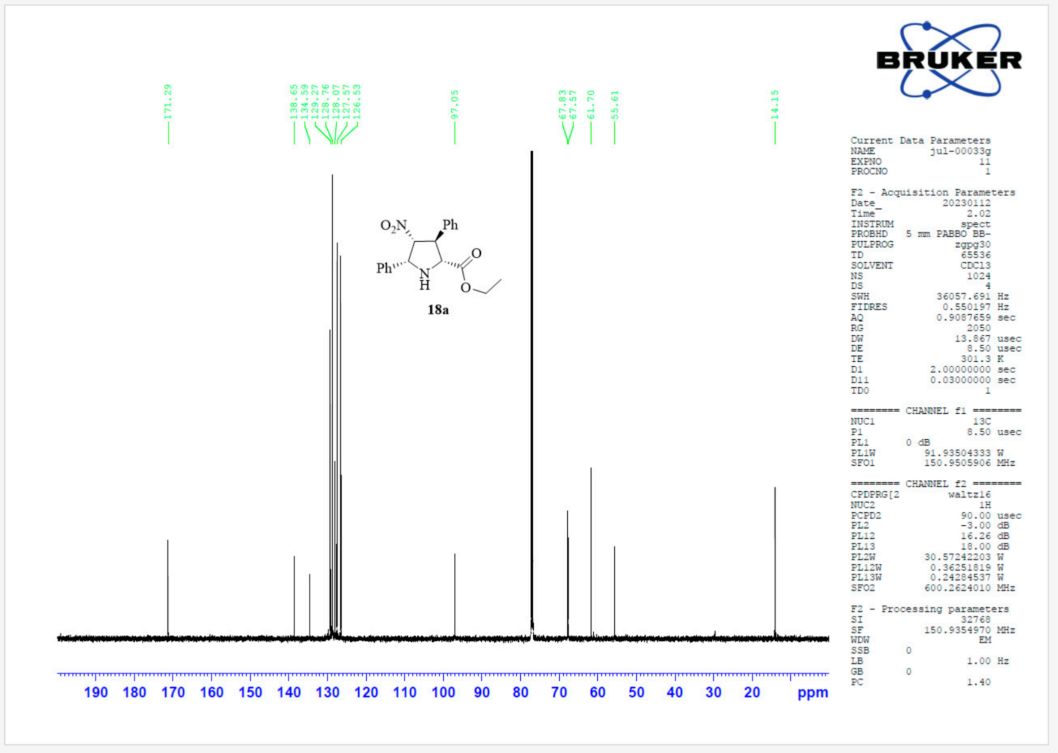

The ^{1}H and ^{13}C NMR of **16b**

^{1}H NMR (600 MHz, $CDCl_3$):

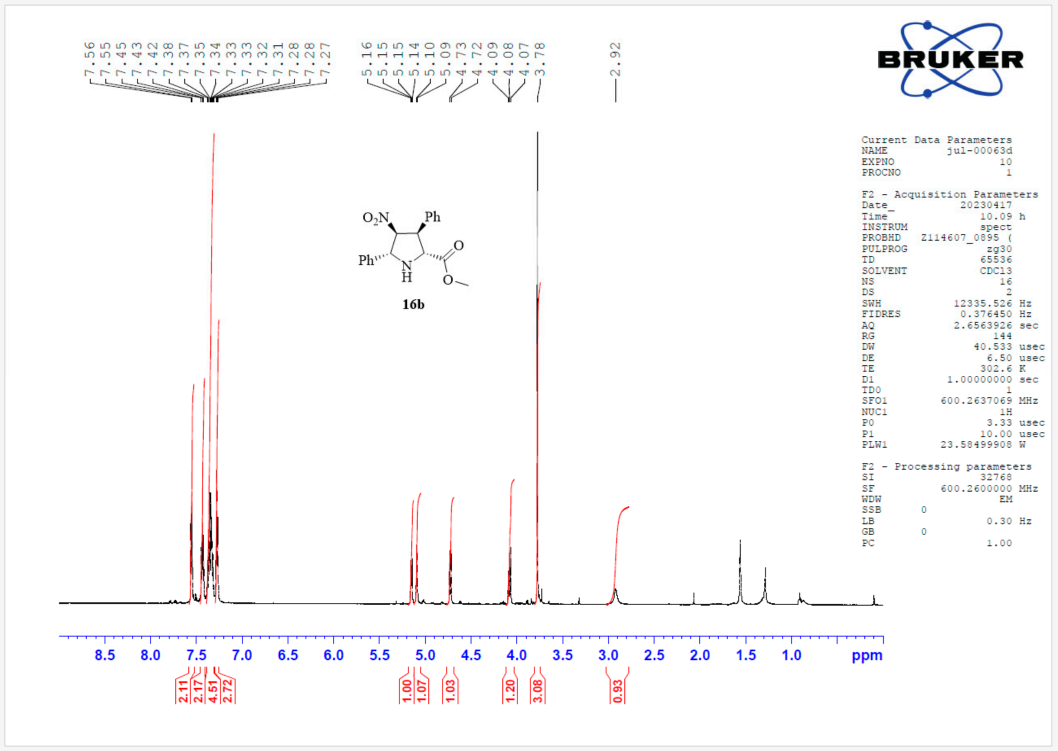

^{13}C NMR (150 MHz, $CDCl_3$):

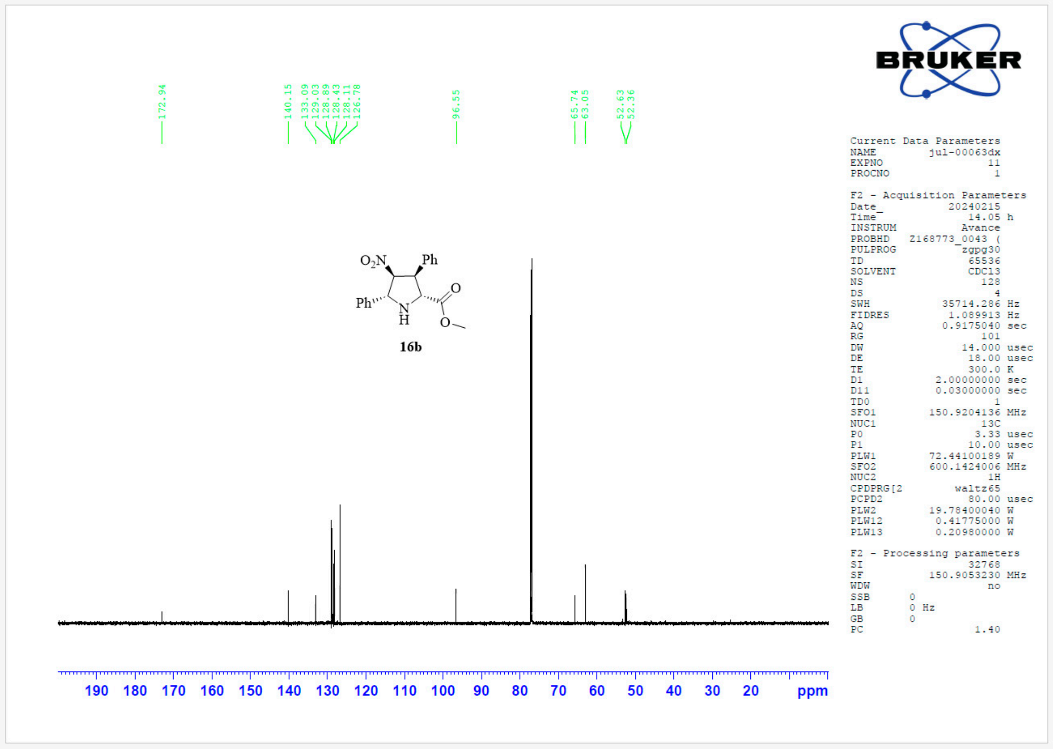

The ^{1}H and ^{13}C NMR of **17b**

^{1}H NMR (600 MHz, $CDCl_3$):

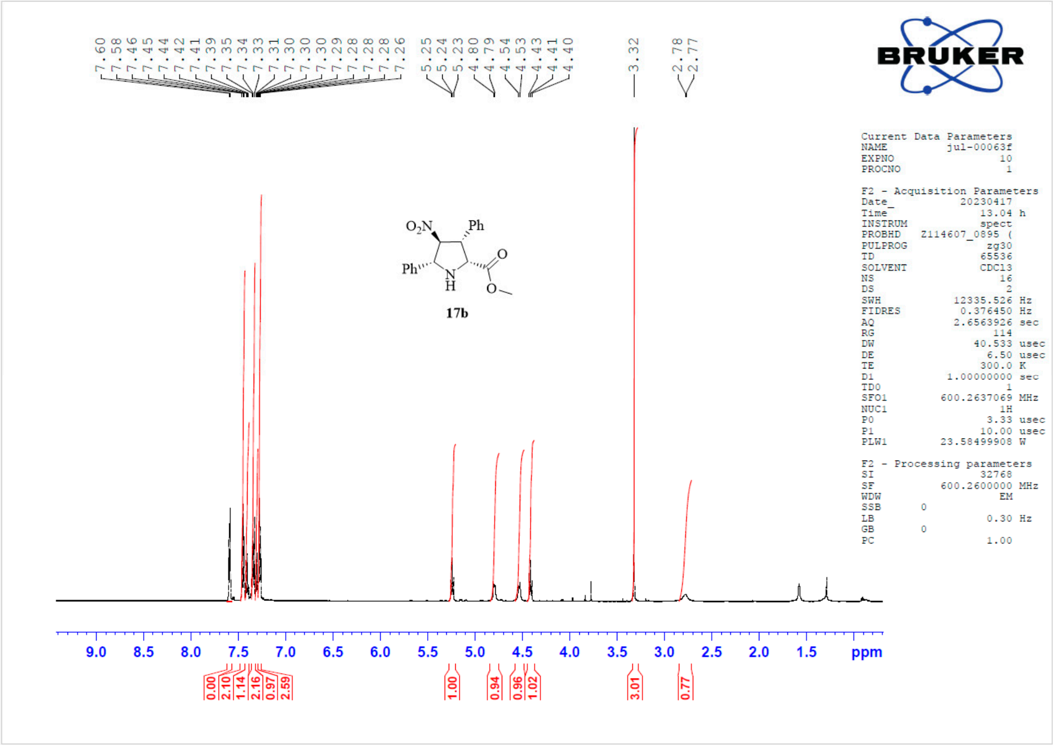

^{13}C NMR (150 MHz, $CDCl_3$):

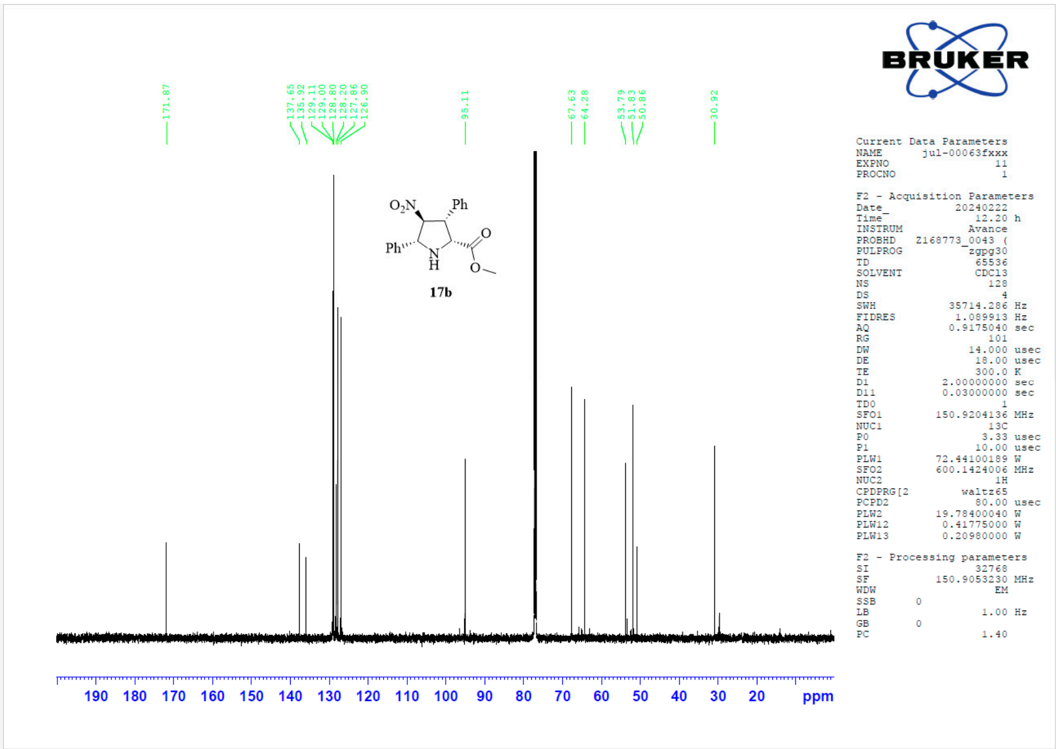

The ^{1}H and ^{13}C NMR of **16c**

^{1}H NMR (600 MHz, $CDCl_3$):

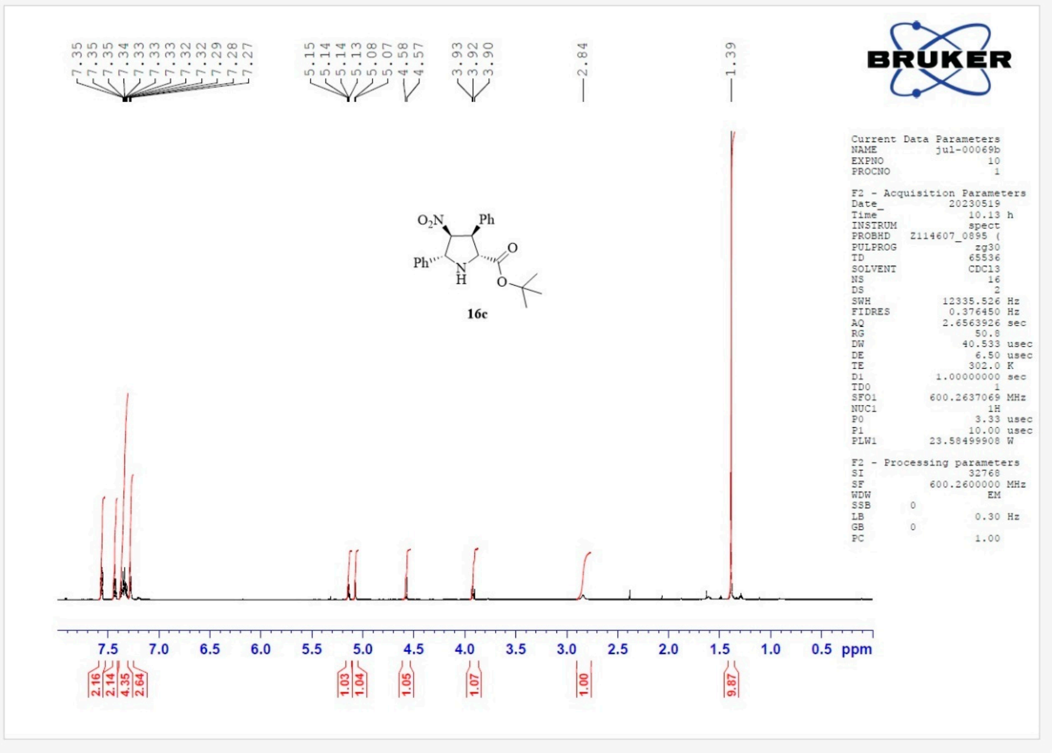

^{13}C NMR (150 MHz, $CDCl_3$):

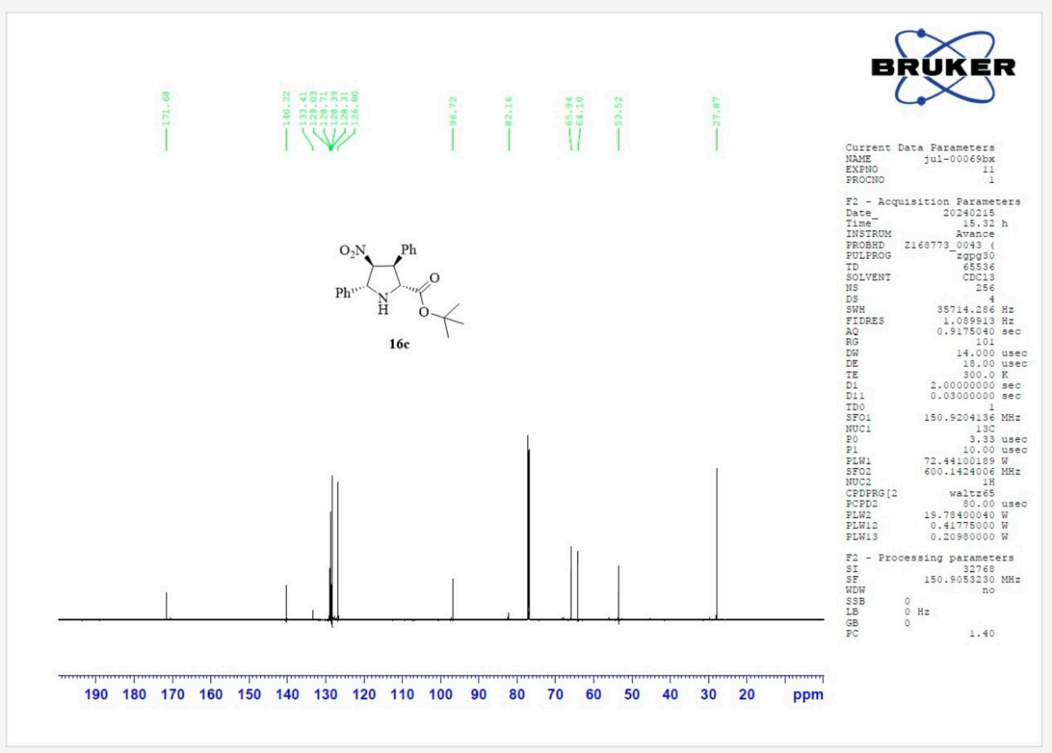

The ^{1}H and ^{13}C NMR of **17c**

^{1}H NMR (600 MHz, $CDCl_3$):

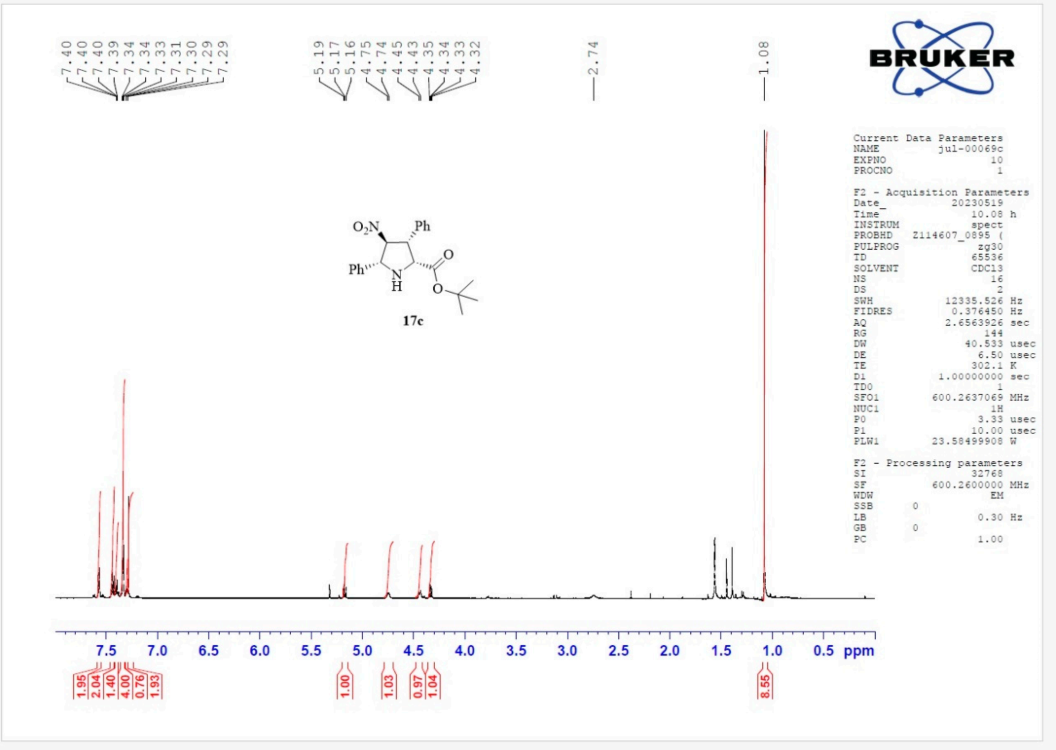

^{13}C NMR (150 MHz, $CDCl_3$):

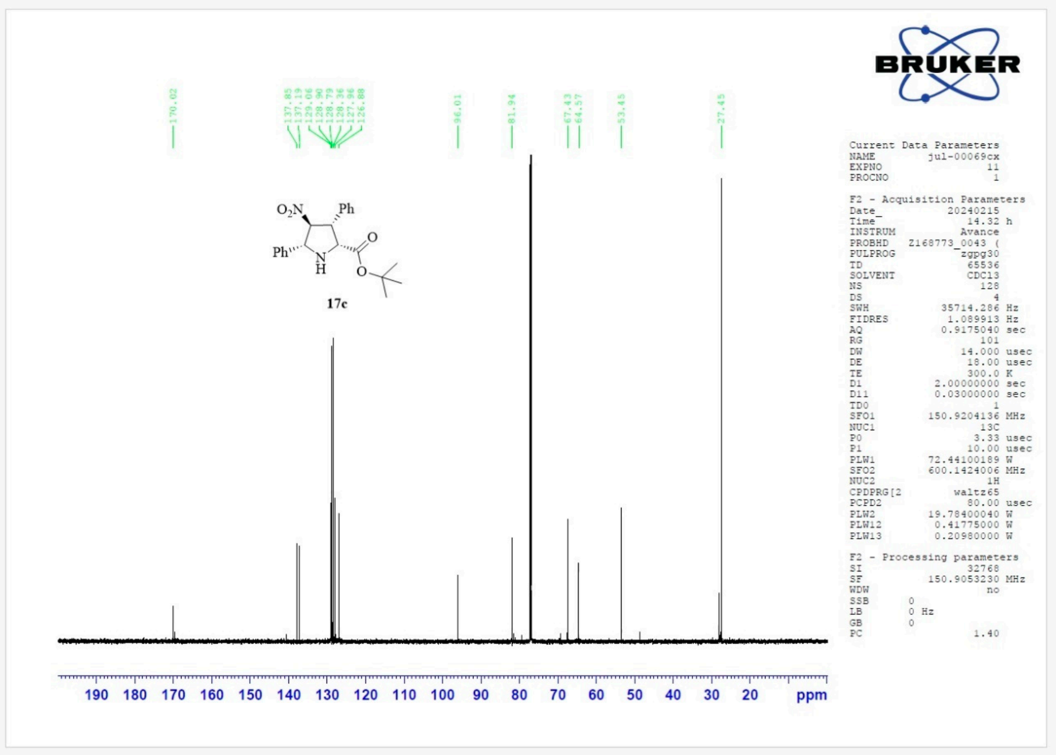

The ^{1}H and ^{13}C NMR of **18c**

^{1}H NMR (600 MHz, $CDCl_3$):

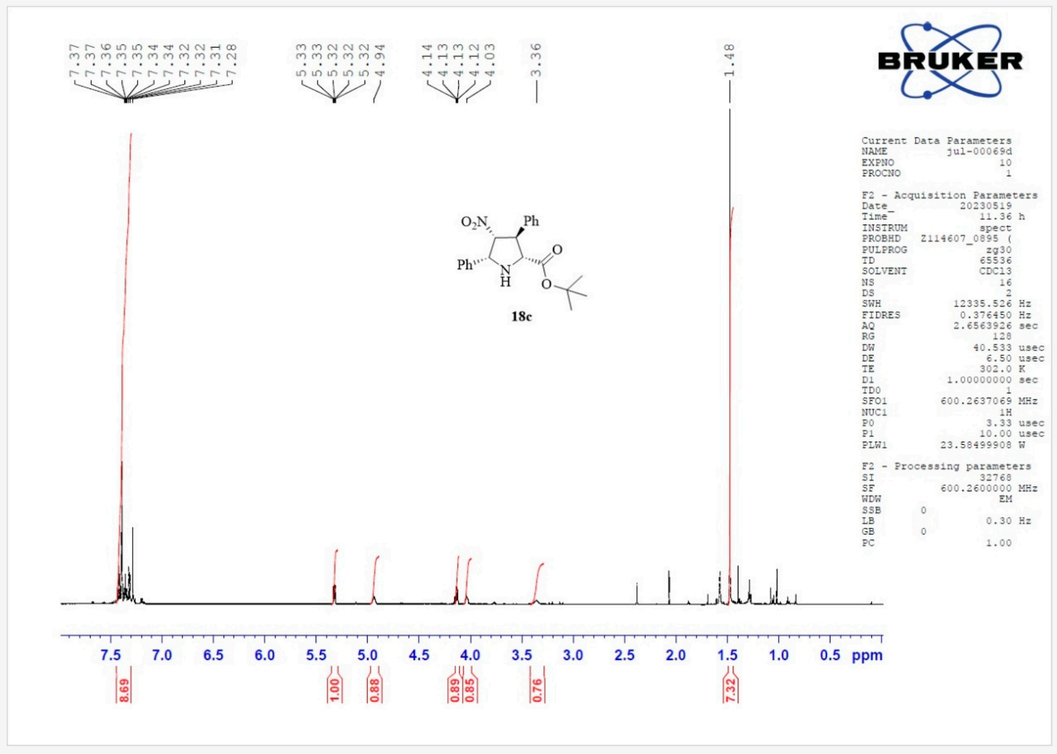

^{13}C NMR (150 MHz, $CDCl_3$):

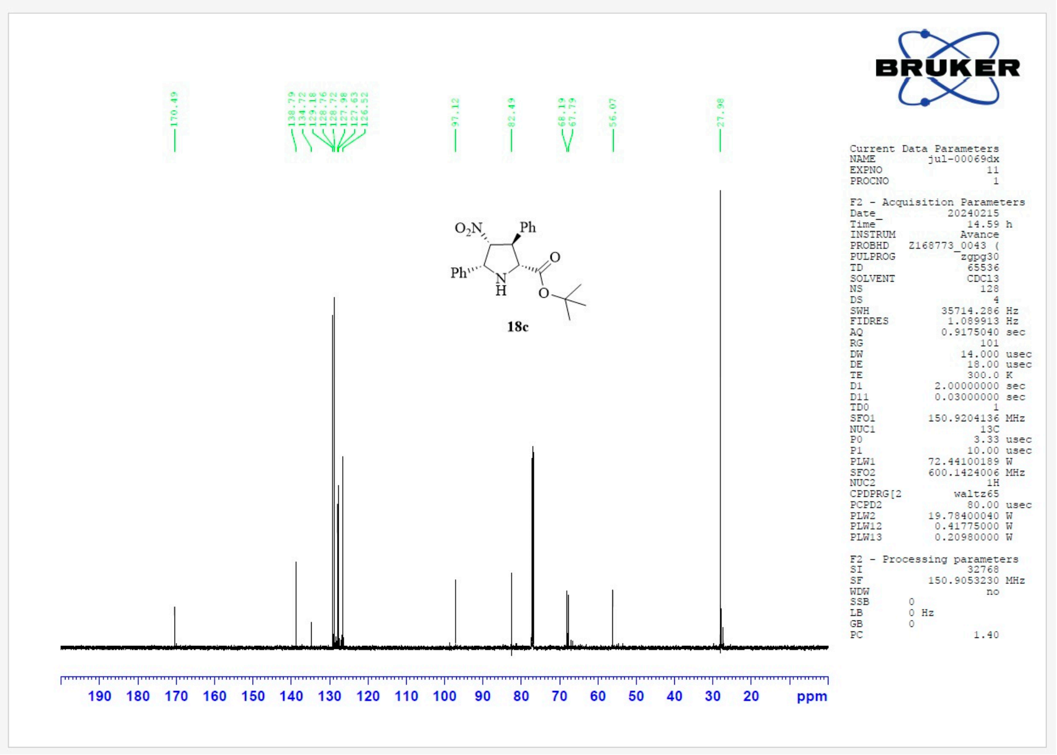

2. HPLC chromatograms

(2*R*,3*S*,4*S*,5*R*)-Ethyl 4-Nitro-3,5-diphenylpyrrolidine-2-carboxylate (*4-epi-endo*) **16a**

ee (95%) determined by HPLC analysis: Chiralcel OD-H column, Hexane : ^{i}PrOH = 90:10, flow = 1.0 mL/min, λ = 254 nm, retention times (min): 39.2 (major), 46.8 (minor).

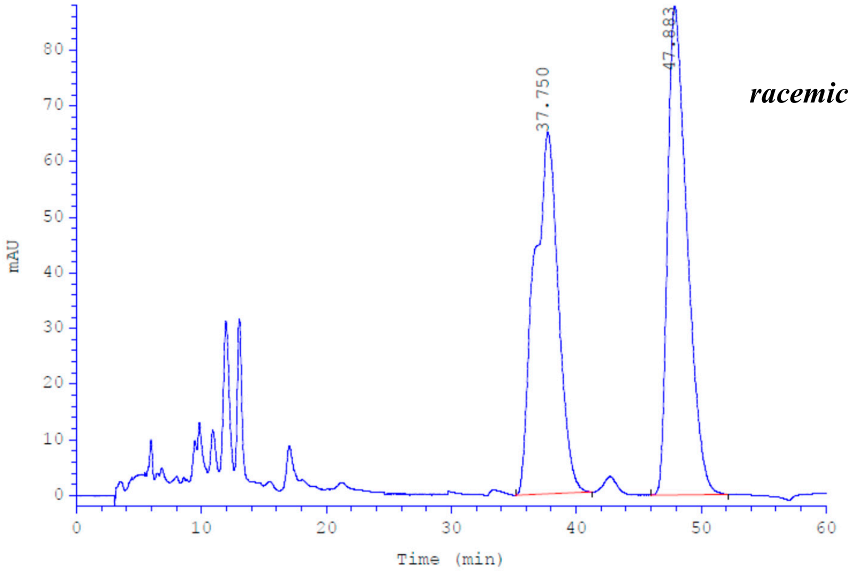

	Ret.time [min]	Start [min]	End [min]	Compound	Amount	Units	Area [mAU*min]	Height [mAU]	% Area	Width [min]	Type
1	37.750	35.17	41.30		0		145.739	64.9135	47.8549	2.358	BB
2	47.883	46.02	52.18		0		158.805	87.8865	52.1451	1.686	BB

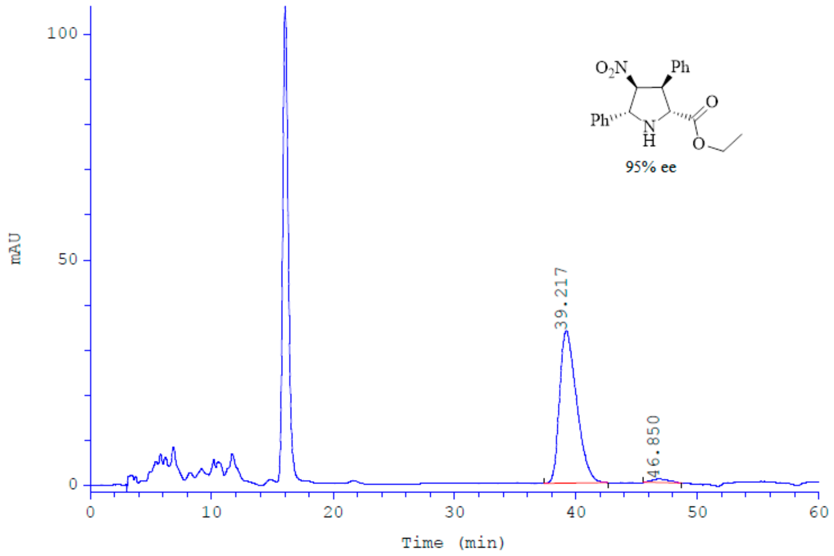

	Ret.time [min]	Start [min]	End [min]	Compound	Amount	Units	Area [mAU*min]	Height [mAU]	% Area	Width [min]	Type
1	39.217	37.38	42.63		0		54.9899	33.8001	97.5331	1.513	BB
2	46.850	45.59	48.73		-1		1.39087	0.839868	2.4669	1.641	BB+

(2*R*,3*R*,4*S*,5*R*)- Ethyl 4-Nitro-3,5-diphenylpyrrolidine-2-carboxylate (*exo*) **17a**

ee (99%) determined by HPLC analysis: Chiralcel OD-H column, Hexane : ^{i}PrOH = 90:10, flow = 1.0 mL/min, λ = 254 nm, retention times (min): 32.9 (minor), 42.2 (major).

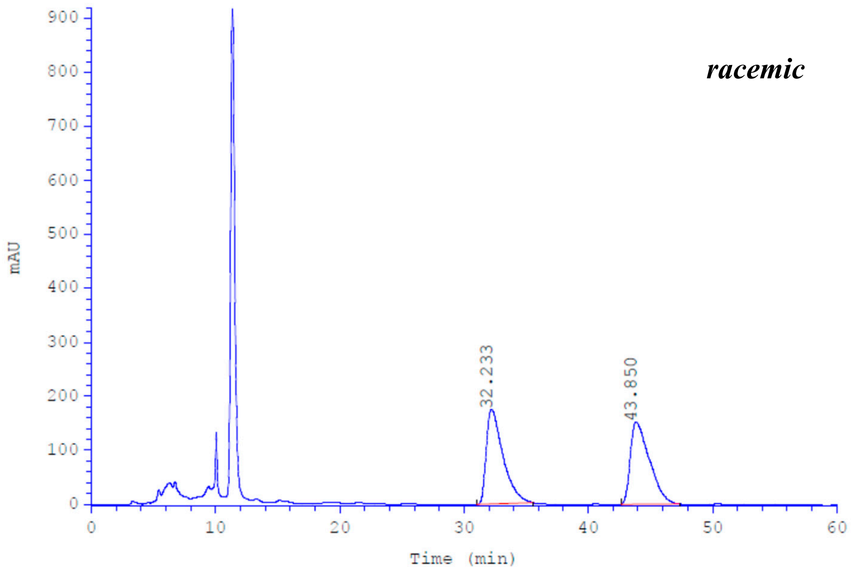

	Ret.time [min]	Start [min]	End [min]	Compound	Amount	Units	Area [mAU*min]	Height [mAU]	% Area	Width [min]	Type
1	32.233	31.05	35.59		-1		273.815	175.718	50.2509	1.391	BB+
2	43.850	42.66	47.33		-1		271.081	152.259	49.7491	1.711	BB+

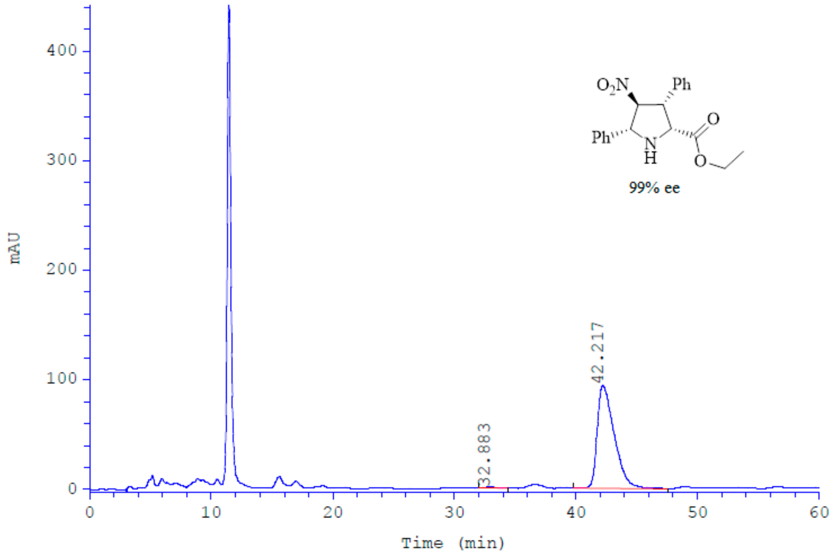

	Ret.time [min]	Start [min]	End [min]	Compound	Amount	Units	Area [mAU*min]	Height [mAU]	% Area	Width [min]	Type
1	32.883	31.99	34.45		-1		1.06041	0.920395	0.6873	1.135	BB+
2	42.217	39.80	47.57		0		153.231	94.0342	99.3127	1.484	BB

(2*R*,3*S*,4*R*,5*R*)- Ethyl 4-Nitro-3,5-diphenylpyrrolidine-2-carboxylate (*endo*) **18a**

ee (1%) determined by HPLC analysis: Chiralcel OD-H column, Hexane : ^{i}PrOH = 90:10, flow = 1.0 mL/min, λ = 254 nm, retention times (min): 32.5 (major), 43.6 (minor).

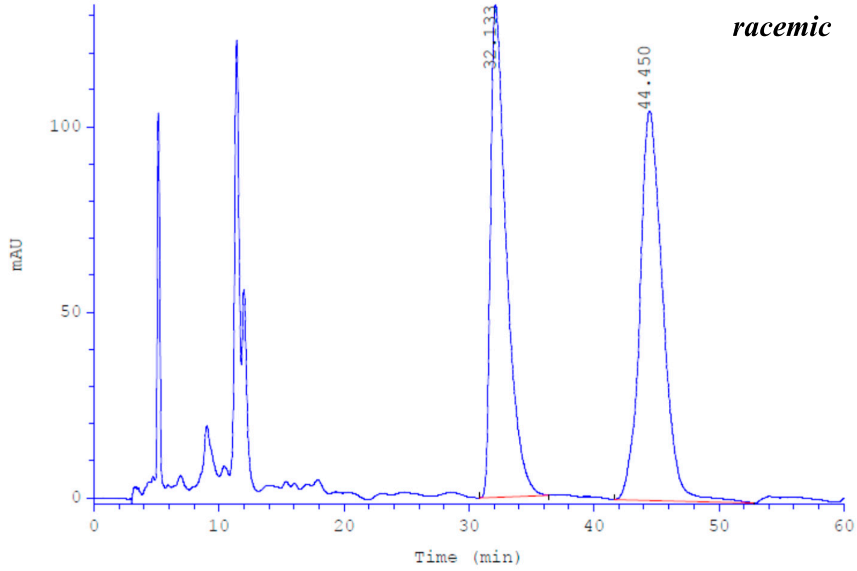

	Ret.time [min]	Start [min]	End [min]	Compound	Amount	Units	Area [mAU*min]	Height [mAU]	% Area	Width [min]	Type
1	32.133	30.87	36.42		0		195.835	132.827	46.6754	1.300	BB
2	44.450	41.65	52.78		0		223.733	105.061	53.3246	1.892	BB

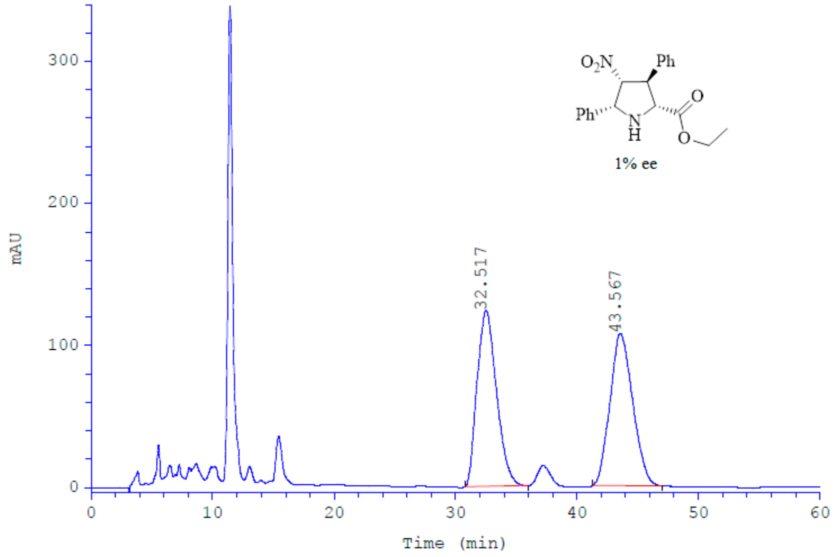

	Ret.time [min]	Start [min]	End [min]	Compound	Amount	Units	Area [mAU*min]	Height [mAU]	% Area	Width [min]	Type
1	32.517	30.78	35.99		-1		229.755	123.791	49.6830	1.785	BB+
2	43.567	41.32	46.99		-1		232.687	107.314	50.3170	2.029	BB+

(2*R*,3*S*,4*S*,5*R*)-Methyl 4-Nitro-3,5-diphenylpyrrolidine-2-carboxylate (*4-epi-endo*) **16b**

ee (29%) determined by HPLC analysis: Chiralcel OD-H column, Hexane : ^{i}PrOH = 90:10, flow = 1.0 mL/min, λ = 254 nm, retention times (min): 11.5 (minor), 17.9 (major).

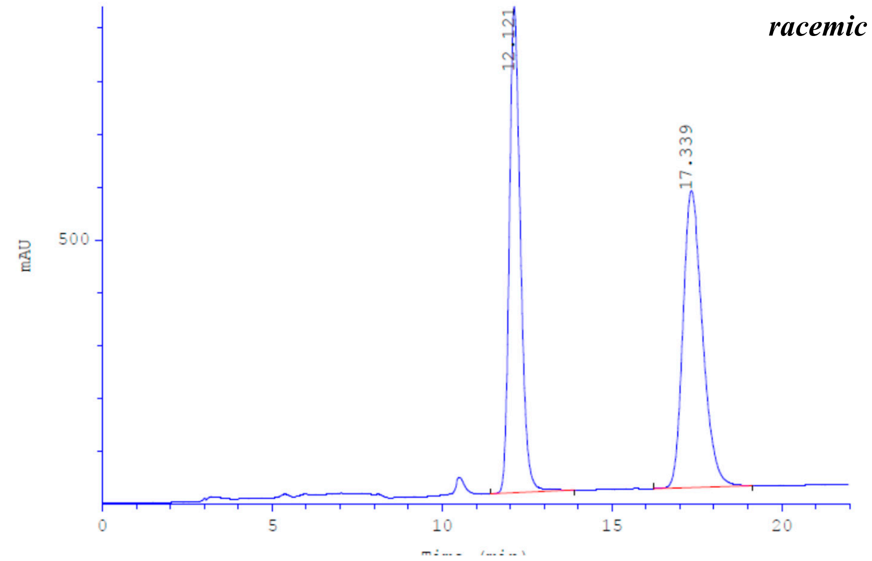

	Ret.time [min]	Start [min]	End [min]	Compound	Amount	Units	Area [mAU*min]	Height [mAU]	% Area	Width [min]	Type
1	12.121	11.42	13.90		0		350.615	919.297	48.3905	0.345	BB
2	17.339	16.24	19.14		0		373.938	561.96	51.6095	0.611	BB

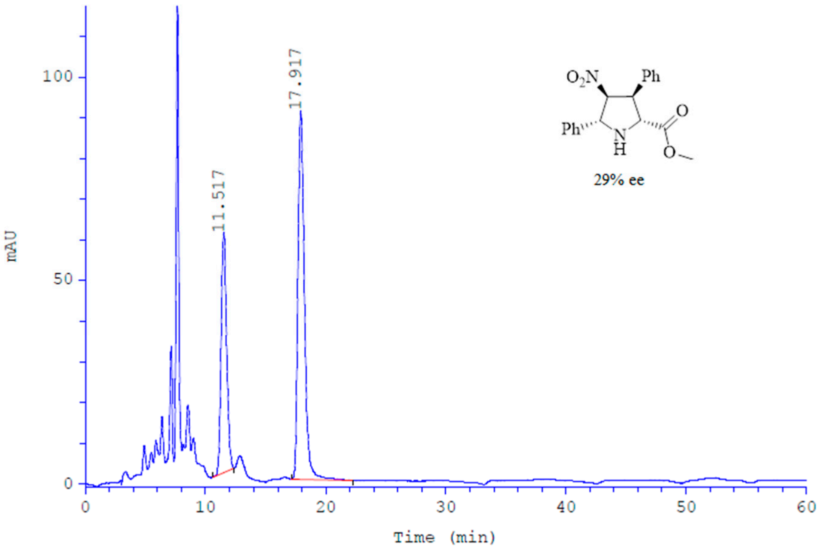

	Ret.time [min]	Start [min]	End [min]	Compound	Amount	Units	Area [mAU*min]	Height [mAU]	% Area	Width [min]	Type
1	11.517	10.62	12.37		0		30.9541	59.0456	35.4339	0.482	BB
2	17.917	17.13	22.25		0		56.4034	90.6417	64.5661	0.551	BB

(2*R*,3*R*,4*S*,5*R*)-Methyl 4-Nitro-3,5-diphenylpyrrolidine-2-carboxylate (*exo*) **17b**

ee (71%) determined by HPLC analysis: Chiralcel OD-H column, Hexane : ^{i}PrOH = 90:10, flow = 1.0 mL/min, λ = 254 nm, retention times (min): 18.4 (major), 29.5 (minor).

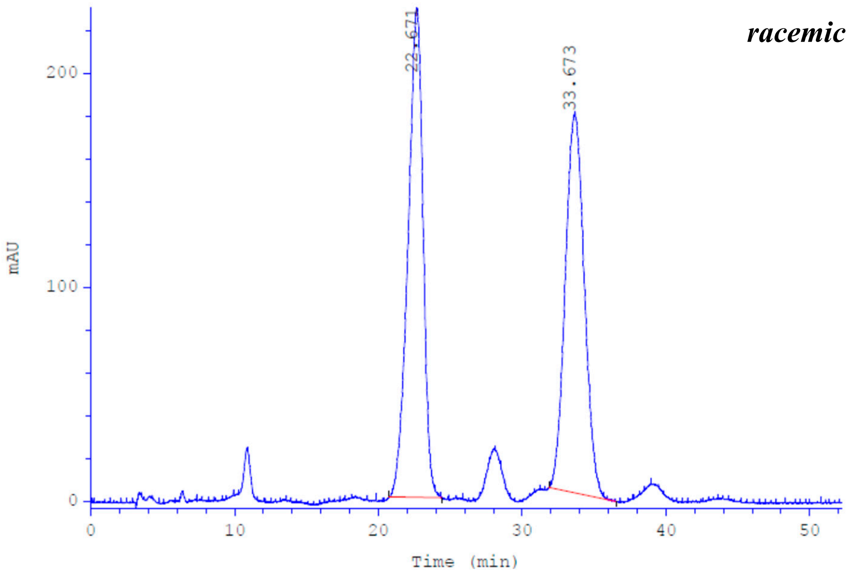

	Ret.time [min]	Start [min]	End [min]	Compound	Amount	Units	Area [mAU*min]	Height [mAU]	% Area	Width [min]	Type
1	22.671	20.74	24.40		-1		276.407	228.727	50.3573	1.120	BB+
2	33.673	31.88	36.53		-1		272.485	178.233	49.6427	1.450	BB+

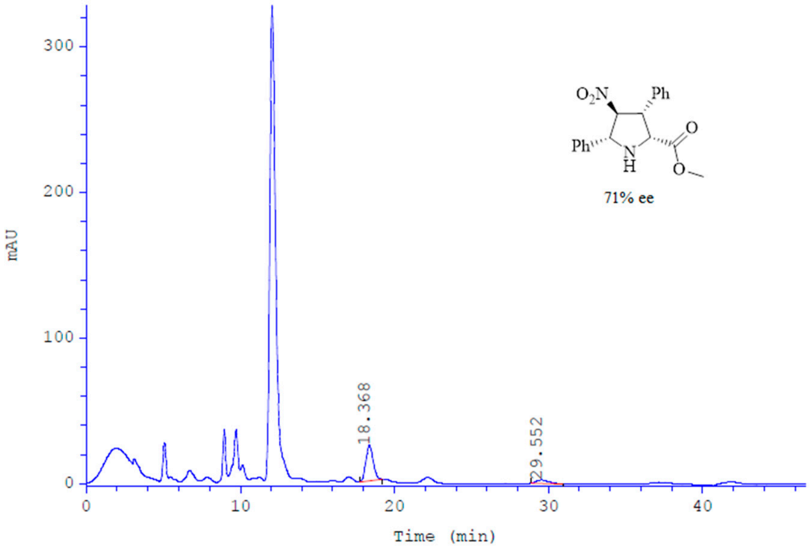

	Ret.time [min]	Start [min]	End [min]	Compound	Amount	Units	Area [mAU*min]	Height [mAU]	% Area	Width [min]	Type
1	18.368	17.75	19.23		0		13.3866	24.1825	85.4035	0.516	BB
2	29.552	28.88	30.96		-1		2.28794	2.2623	14.5965	1.007	BB+

(2*R*,3*S*,4*S*,5*R*)-*Tert*-butyl 4-Nitro-3,5-diphenylpyrrolidine-2-carboxylate (*4-epi-endo*) **16c**

ee (>1%) determined by HPLC analysis: Chiralcel OD-H column, Hexane : ^{i}PrOH = 90:10, flow = 1.0 mL/min, λ = 254 nm, retention times (min): 12.3, 13.1.

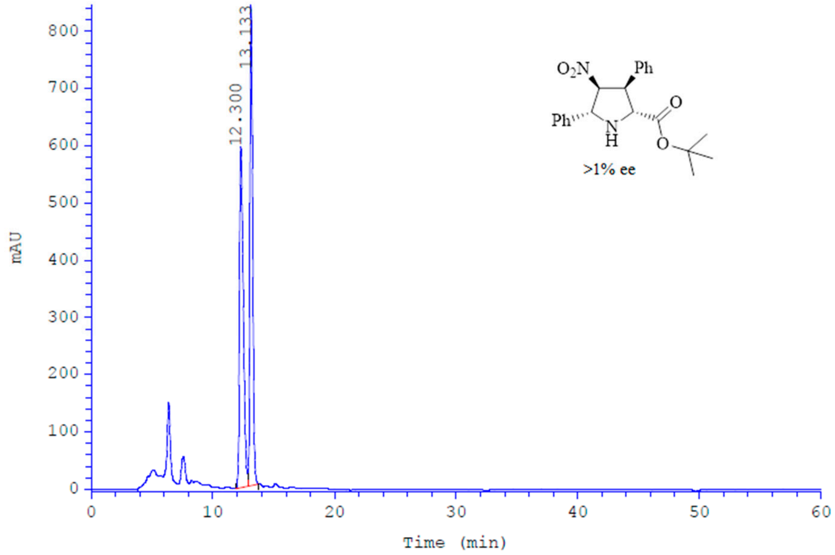

	Ret.time [min]	Start [min]	End [min]	Compound	Amount	Units	Area [mAU*min]	Height [mAU]	% Area	Width [min]	Type
1	12.300	11.88	12.85		0		218.559	595.778	49.7338	0.338	BP
2	13.133	12.85	13.72		0		220.898	839.701	50.2662	0.246	PB

(2*R*,3*R*,4*S*,5*R*)-*Tert*-butyl 4-Nitro-3,5-diphenylpyrrolidine-2-carboxylate (*exo*) **17c**

ee (48%) determined by HPLC analysis: Chiralcel OD-H column, Hexane : ^{i}PrOH = 90:10, flow = 1.0 mL/min, λ = 254 nm, retention times (min): 12.7 (minor), 14.1 (major).

racemic

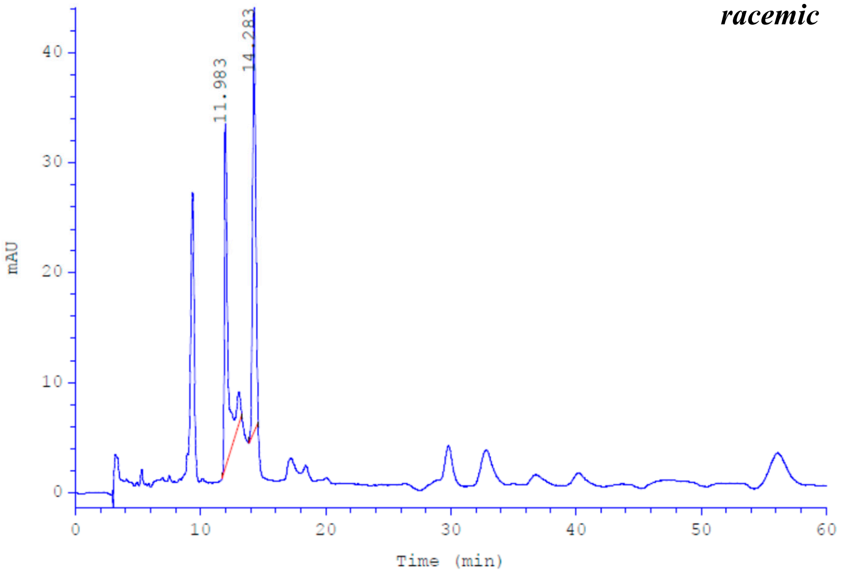

	Ret.time [min]	Start [min]	End [min]	Compound	Amount	Units	Area [mAU*min]	Height [mAU]	% Area	Width [min]	Type
1	11.983	11.71	13.29		-1		10.0194	31.1951	47.6487	0.210	BB+
2	14.283	13.88	14.61		-1		11.0082	38.3994	52.3513	0.272	BB+

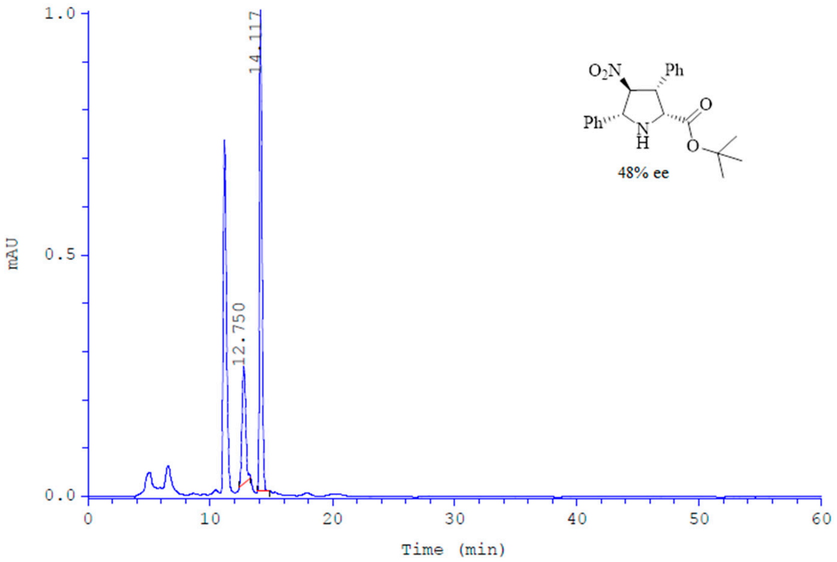

	Ret.time [min]	Start [min]	End [min]	Compound	Amount	Units	Area [mAU*min]	Height [mAU]	% Area	Width [min]	Type
1	12.750	12.37	13.31		-1		84.5235	243.073	25.9290	0.320	BB+
2	14.117	13.84	14.91		-1		241.457	996.327	74.0710	0.229	BB+

(2*R*,3*S*,4*R*,5*R*)-*Tert*-butyl 4-Nitro-3,5-diphenylpyrrolidine-2-carboxylate (*endo*) **18c**

ee (11%) determined by HPLC analysis: Chiralcel OD-H column, Hexane : ^{i}PrOH = 90:10, flow = 1.0 mL/min, λ = 254 nm, retention times (min): 16.6 (major), 29.9 (minor).

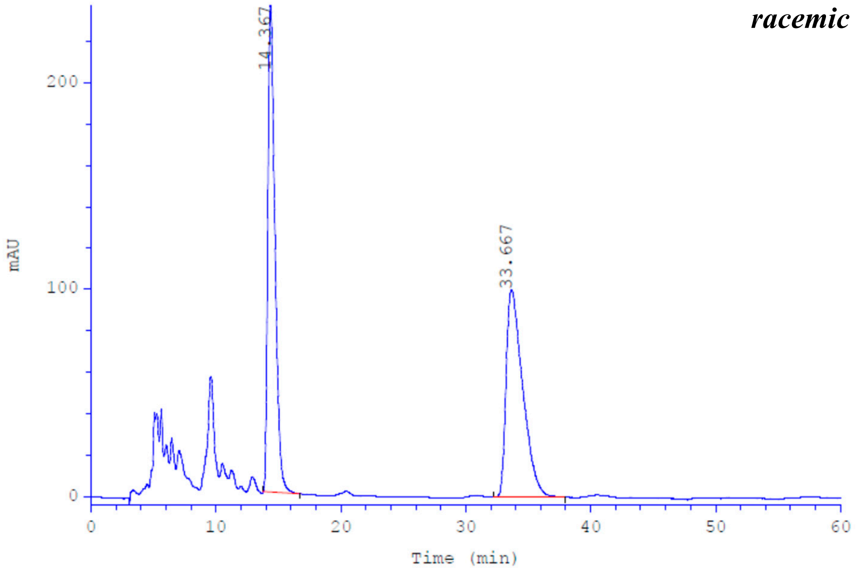

	Ret.time [min]	Start [min]	End [min]	Compound	Amount	Units	Area [mAU*min]	Height [mAU]	% Area	Width [min]	Type
1	14.367	13.78	16.71		-1		154.691	234.969	50.0046	0.603	BB+
2	33.667	32.27	37.95		0		154.663	100.132	49.9954	1.432	BB

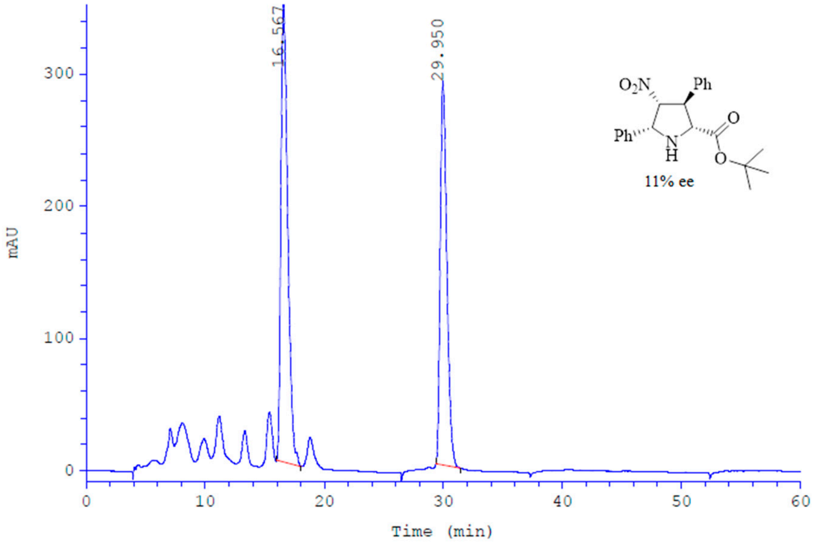

	Ret.time [min]	Start [min]	End [min]	Compound	Amount	Units	Area [mAU*min]	Height [mAU]	% Area	Width [min]	Type
1	16.567	15.98	18.04		-1		232.172	345.385	55.3214	0.628	BB+
2	29.950	29.38	31.45		-1		187.507	290.122	44.6786	0.604	BB+
